# Genetic Improvement in Plant Architecture, Maturity Duration and Agronomic Traits of Three Traditional Rice Landraces through Gamma Ray-Based Induced Mutagenesis

**DOI:** 10.3390/plants11243448

**Published:** 2022-12-09

**Authors:** Richa Sao, Parmeshwar K. Sahu, Raviraj Singh Patel, Bikram K. Das, Ljupcho Jankuloski, Deepak Sharma

**Affiliations:** 1 Department of Genetics and Plant Breeding, Indira Gandhi Krishi Vishwavidyalaya (IGKV), Raipur 492012, India; 2 Nuclear Agriculture and Biotechnology Division, Bhabha Atomic Research Centre (BARC), Mumbai 400085, India; 3 Plant Breeding and Genetics Section, Joint FAO/IAEA Centre, International Atomic Energy Agency, 1400 Vienna, Austria

**Keywords:** rice landraces, mutation breeding, genetic improvement, gamma rays, plant architecture, maturity duration, SSR markers, genomic similarity

## Abstract

Mutation breeding offers a simple, fast and efficient way to rectify major defects without altering their original identity. The present study deployed radiation (gamma rays @ 300Gy)-induced mutation breeding for the improvement and revival of three traditional rice landraces, viz., Samundchini, Vishnubhog and Jhilli. Among the various putative mutants identified in the M2 generation, only three, ten and five rice mutants of Samundchini, Vishnubhog and Jhilli, respectively, were advanced to the M4, M5 and M6 generations, along with their parents and three checks for evaluations based on 13 agro-morphological and 16 grain quality traits. Interestingly, all the mutants of the three landraces showed a reduction in days to 50% flowering and plant height as compared to their parents in all the three generations. The reduction in days to 50% flowering ranges from 4.94% (Vishnubhog Mutant V-67) to 21.40% (Jhilli Mutant J-2-13), whereas the reduction in plant height varies from 11.28% (Vishnubhog Mutant V-45-2, Vishnubhog Mutant V-67) to 37.65% (Jhilli Mutant J-15-1). Furthermore, two, six and three mutants of Samundchini, Vishnubhog and Jhilli have increased their yield potential over their corresponding parents, respectively. Interestingly, Samundchini Mutant S-18-1 (22.45%), Vishnubhog Mutant V-74-6 (36.87%) and Jhilli Mutant J-13-5 (25.96%) showed the highest yield advantages over their parents. Further, a pooled analysis of variance based on a randomized complete block design revealed ample variations among the genotypes for the studied traits. In addition, all the traits consistently showed high to moderate PCV and GCV and a slight difference between them in all three generations indicated the negligible effect of the environment. Moreover, in the association analysis, the traits, viz., fertile spikelets/panicle, panicle length, total tillers/plant, spikelet fertility percent and 100-seed weight showed the usual grain yield/plant, whereas the traits hulling (%) and milling (%) with HRR (%) consistently showed high direct effects and significant positive correlations. The SSR marker-based genome similarity in rice mutants and corresponding parents ranged from 95.60% to 71.70% (Vishnubhog); 95.62% to 89.10% (Samundchini) and 95.62% to 80.40% (Jhilli), indicating the trueness of the mutants. Moreover, the UPGMA algorithm and Gower distance-based dendrogram, neighbour joining tree and PCA scatter diagram assured that mutants were grouped with their respective parents and fell into separate clusters showing high similarity between mutants and parents and dissimilarity among the 24 genotypes. Overall, the information and materials generated from the current study will be very useful and informative for students, researchers and plant breeders. Additionally, our results also showed that irradiation could generate a considerable amount of genetic variability and provide new avenues for crop improvement and diversification.

## 1. Introduction

Rice (*Oryza sativa* L.) is the most important cereal crop and staple food for more than half of the world’s population [1]. It is popularly called a ‘global grain’, as it can adapt to a wide range of environmental conditions and, thus, grow in more than 100 countries [2,3]. The swelling human population has set off an alarm to amplify food production and productivity in limited arable lands that are decreasing day by day due to urbanisation and industrialisation. Additionally, it is estimated that food production should be at least doubled by the year 2050 in order to meet the needs of a constantly rising population [1,3,4,5]. Therefore, efforts to increase rice productivity are an area of recent research.

India is known for its rich heritage of indigenous rice landraces, which are adapted in wide agro-ecosystems. Presently, the Chhattisgarh State of India has more than 23,250 accessions of rice, including 210 wild species, accounting for the largest in the country and second largest in the world after the International Rice Research Institute (IRRI), Manila, the Philippines [6,7]. Rice landraces are rich sources of several valuable and useful genes for yield-attributing traits: resistance against biotic and abiotic stresses, resistant to herbicides; rich in micronutrient content, a wider range of adaptation, etc. that can be used in rice improvement programmes [8]. Apart from many useful attributes owned by these landraces, they have some lacunae, viz., low-yield potential, tall plant stature, late maturity duration, photoperiod sensitivity, lodging, grain-shattering habit, open plant canopy, etc. that makes them unfit for commercial cultivation. Moreover, with the development of high-yielding varieties and hybrids, areas of cultivation of these landraces have been significantly marginalized. It is awful to say that many of them have been extricated from farmers’ fields, causing a severe threat to rice biodiversity. Such problems need to be addressed carefully in a sustainable manner.

To overcome the same, scientists are working on the improvement of rice landraces through various breeding, biotechnological and genomic approaches [9]. However, most of these methods have significantly altered the genetic makeup of the genotypes by changing their original grain qualities, which has restricted their wider acceptability among farmers. In this regard, mutation breeding offers a simple, cost-effective and powerful means of inducing genetic variations followed by trait selection to improve one or two major defects without altering the genuine characters of the plants [10]. Moreover, the developed mutant varieties enhance the crop biodiversity and offer useful breeding materials for further crop improvements [10,11]. Until now, about 3406 mutant varieties in more than 225 crops have been developed through induced mutagenesis worldwide and have been registered in the FAO/IAEA Mutant Variety Database (MVD), International Atomic Energy Agency, Vienna, Austria [12]. The Asian countries, particularly China (817), Japan (479) and India (345), have developed about 50% of the total crop mutant varieties in the world. Interestingly, the highest numbers of mutants have been developed in 852 rice crops in the world and 64 in India [12].

The success of mutation breeding largely depends on the use of appropriate mutagens, their optimum dose for the induction of desirable mutations, selection and handing of mutagenic populations. Once the dose has been optimized, seeds or plant tissues are irradiated with the optimum dose of desirable mutagens to constitute the M1 population, which, further on, constitutes the M2 population itself for the selection of desirable plant types. Understanding the wide range of genetic parameters and associations among the traits in mutant lines is a prerequisite for exploiting them in breeding programs. Therefore, the characterisations of screened mutants for grain yield-attributing traits and grain quality traits are essential from the M4 generation onward. After the M6 generation, the mutant is considered to be a stable genotype and may be subjected to multilocation trials (MLTs) if they have potential. Thereafter, potential mutants may be released and notified for commercial cultivation in farmers’ fields [13,14].

Alterations in plant traits by mutation breeding is a result of changes in the DNA sequences, which leads to alterations in the gene expression patterns, hence resulting in the appearance of a modified trait. These variations in DNA sequences may be traced with the help of molecular markers by a genomic similarity or dissimilarity study. Revealing the genetic relationships among mutants and their wild types using DNA markers adds more insight to this study. It helps in estimating the extent of the genetic relationship among individuals, ignoring the effect of genotype–environment (GxE) interactions on genotypes, hence making this a robust study. Microsatellites or simple sequence repeat (SSR) markers are reliable and relatively inexpensive, codominant markers with high levels of allelic diversity. In rice, they are abundant, distributed throughout the genome and have been used in genetic diversity studies [15,16,17].

Taking into consideration the aforementioned views, the present investigation was carried out to develop improved mutant lines from three traditional rice landraces through gamma ray-based mutation breeding, their evaluation based on agro-morphological and grain quality parameters and their genomic similarity and diversity study through SSR markers.

## 2. Results and Discussion

### 2.1. Identification and Selection of Desirable Mutants in M2 Generation

Macro-mutants are recognized at the single plant level, and this includes all types of chlorophyll mutations and viable morphological changes in plant characters. In Samundchini, 81 putative macro-mutants were identified, which included chlorophyll (45) and viable morphological (36) mutants. Moreover, in Vishnubhog, 98 putative macro-mutants were obtained, which included chlorophyll (56) and viable morphological (42) mutants. Similarly, in Jhilli, 51 total putative macro-mutants were obtained, which comprised 32 chlorophyll mutants and 19 morphological mutants (Supplementary Table S1). All the viable morphological mutants were considered as putative mutants and harvested separately to advance them in the next generation (M3) for further confirmation.

### 2.2. Confirmation of Mutants in M3 Generation

All the viable morphological mutants selected in the M2 generation were grown as the M3 population to confirm their mutant behaviour. Chlorophyll mutants were not included for further generation advancement study due to the noneconomic use of this trait. Viable morphological mutants deviating from the traits under selection, showing poor yield performance, susceptible to lodging and shattering and poor crop canopy were discarded after evaluation in the M3 generation, whereas mutants depicting true to type, better yield-attributing traits, having good plant stature, possessing uniqueness and academically important in studying the trait behaviour were advanced into the M4 generation for further study. Out of 19, 36 and 42 mutants of Jhilli, Samundchini and Vishnubhog, only five (26.31%), three (8.33%) and ten (23.80%) mutants were selected, respectively, as true to type and superior (Table 1). True to type mutants were bulked and advanced, whereas, in segregating lines, further selections were made for desirable traits. Most of the putative mutants were discarded in this generation.

### 2.3. Performance of True to Type Rice Mutants in M4, M5 and M6 Generations under Replicated Yield Trials

**Figure 1:**
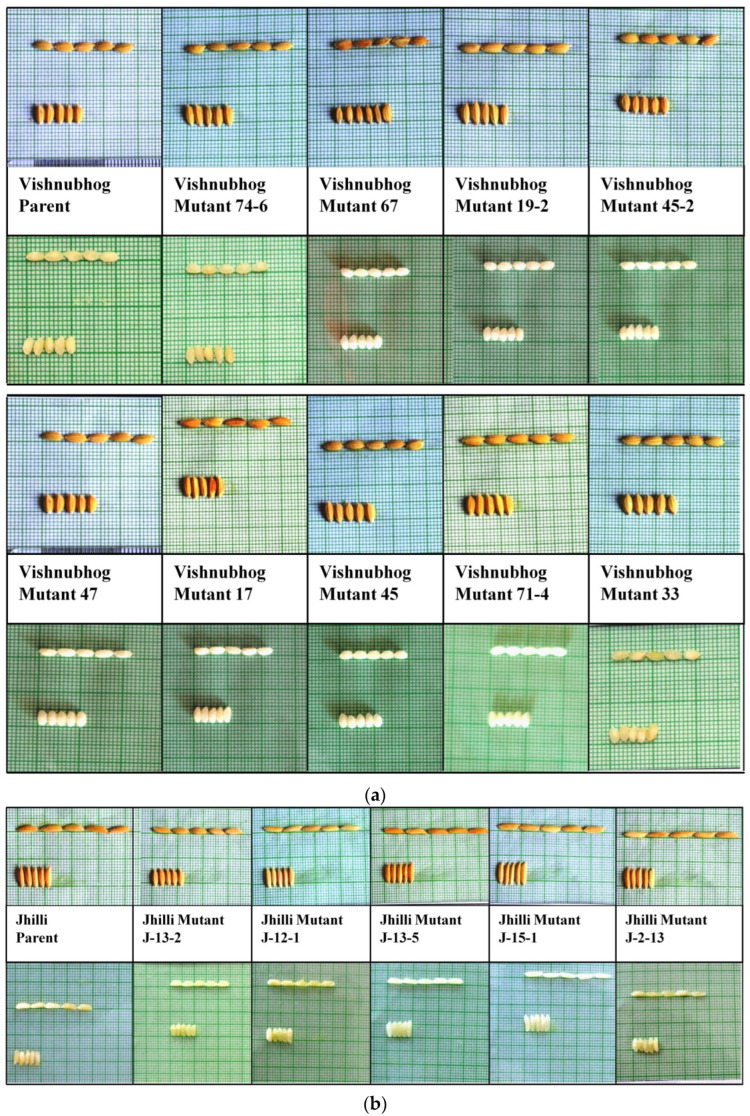

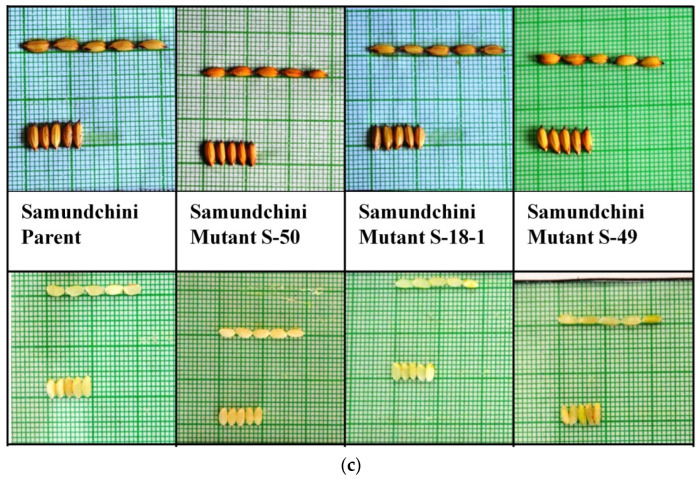
(**a**). Similarity in the lengths and widths of rough paddy and milled rice of Vishnubhog mutants with their corresponding parent Vishnubhog. (**b**). Similarity in the lengths and widths of rough paddy and milled rice of Jhilli mutants with their corresponding parent Jhilli. (**c**). Similarity in the lengths and widths of rough paddy and milled rice of Samundchini mutants with their corresponding parent Samundchini.

The mean performance for the entire agro-morphological and grain quality traits showed similar trends in all the three generations for all the genotypes (Supplementary Tables S2 and S3). Considering the major drawbacks of rice landraces, targeted selection for reduced maturity duration and reduced plant height was performed in the M2 generation in all three landraces. However, all the selected mutants were evaluated for various agro-morphological traits and grain quality traits. Long sunshine hours during Rabi season 2019–20 affected the flower initiation in rice, as some of them were photoperiod-sensitive. It affects the expression patterns of agro-morphological traits, viz., plant growth and maturity duration of crops, and thus caused variations in the mean performance for the traits such as days to 50% flowering, plant height and grain yield per plant. All the mutants of Samundchini, Vishnubhog and Jhilli showed reduced durations of maturity or earliness and reduced plant heights as compared to their corresponding parents in all three seasons. Earliness is advantageous over late maturing genotypes, as it minimises the effect of climate change on farming activities and also ensures a quick economic return on the harvest. Similarly, the reduced height of mutants was advantageous over parents, as it prevented lodging, shattering of grains and had a higher yield over the parents and checks. Rice mutants with early maturity durations and reduced plant heights were also reported by Purwanto et al. [18], Sharma et al. [19] and Roy et al. [20]. While considering the grain yield, two, five and four mutants of Samundchini, Vishnubhog and Jhilli showed higher grain yields over their corresponding parents, respectively. The possible causes of higher yields in mutants over their parents are a reduced height and reduced maturity duration. Photo assimilates in the form of energy engaged for extra plant height and more maturity durations might have been diverted into grains. Higher yields of rice mutants over their parents have also been previously reported by Purwanto et al. [18], Sharma et al. [19], Roy et al. [20], Schiocchet et al. [21] and Kato et al. [22]. Furthermore, the grain quality traits and grain types of mutants were similar to their corresponding parents (Figure 1a–c). In addition, the rest of the traits in the mutant lines were more or less similar to their corresponding parents.

**Figure 2:**
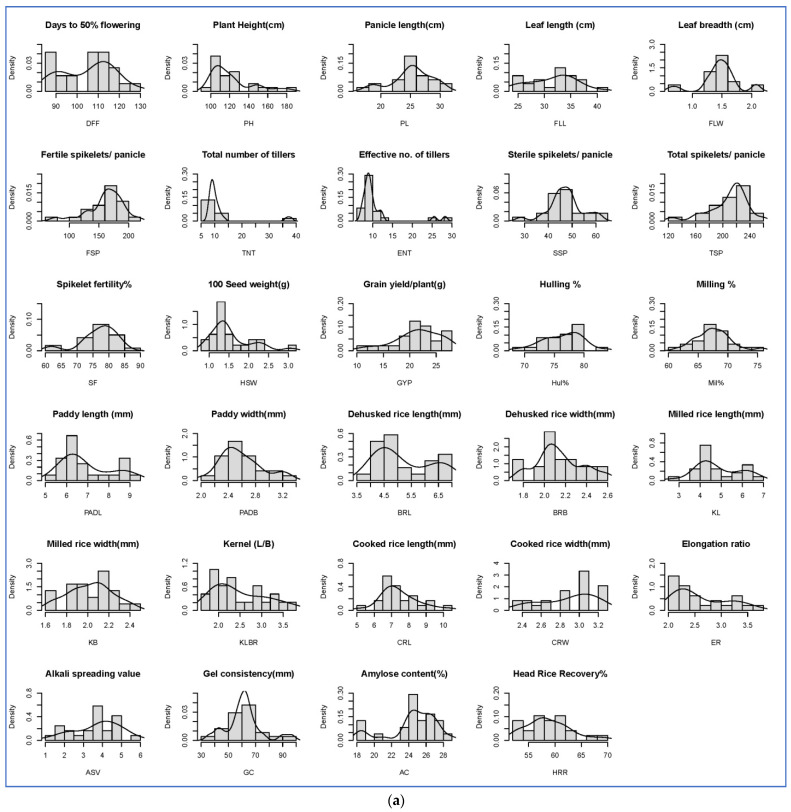

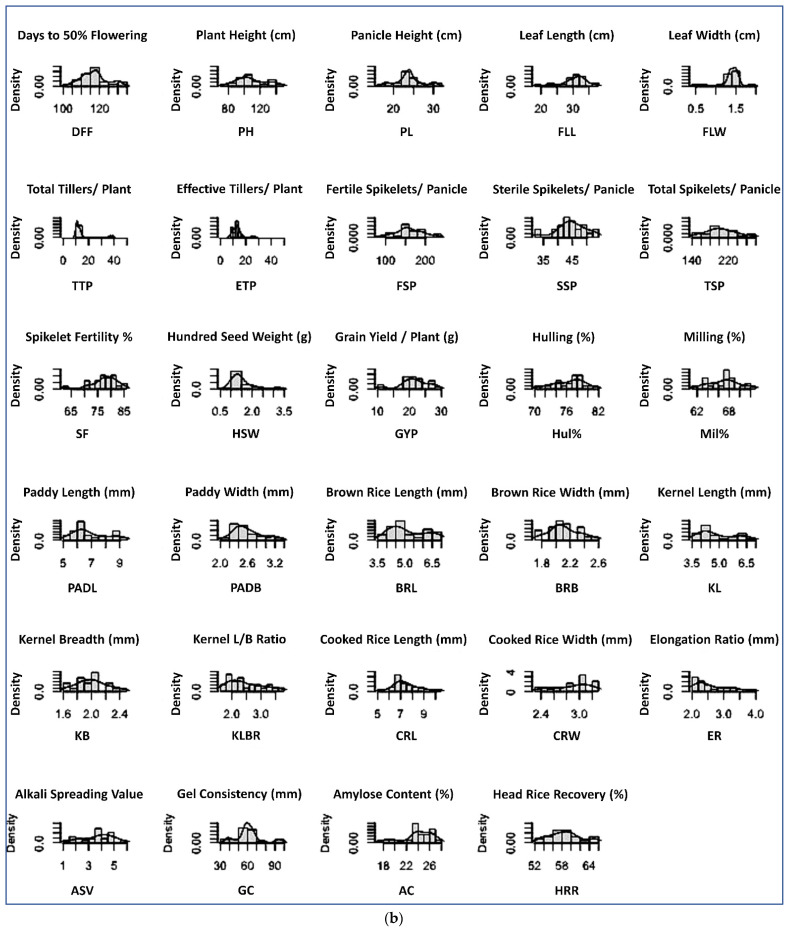

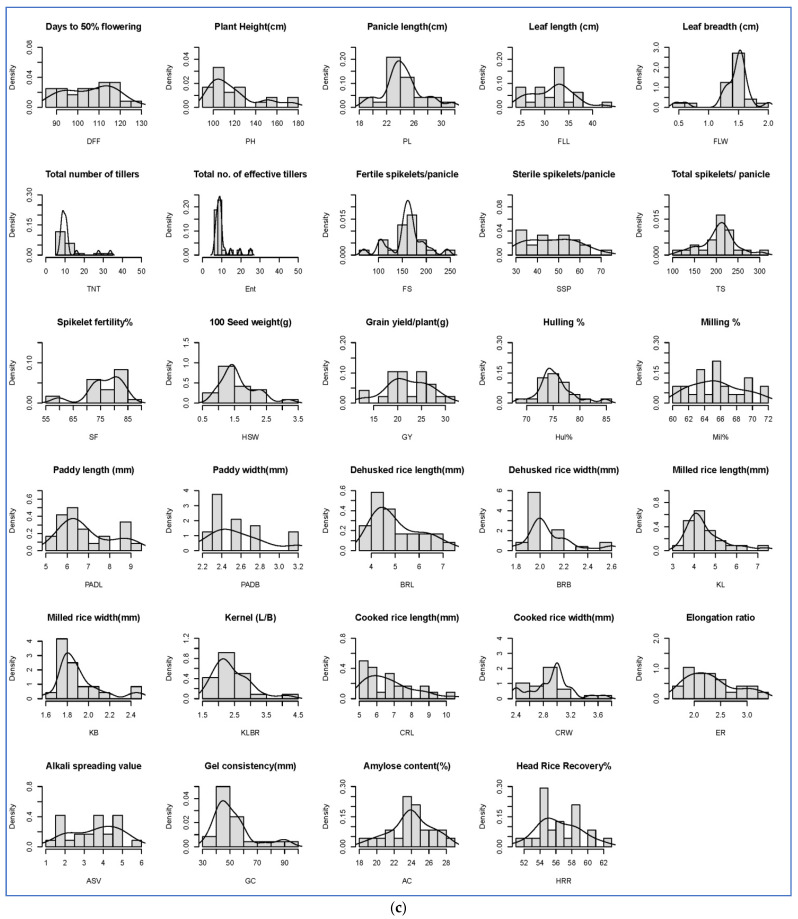
(**a**). Frequency distribution-based histogram for all the traits during the Kharif (wet) season—2019. (**b**). Frequency distribution-based histogram for all the traits during the Rabi (Dry) season—2019–20. (**c**). Frequency distribution-based histogram for all the traits during the Kharif (wet) season—2020.

The frequency distribution-based histogram and Shapiro–Wilk W test for the normal distribution of traits revealed that the traits days to 50% flowering (DFF), plant height (PH), panicle length (PL), flag leaf length (FLL), flag leaf width (FLW), total tillers per plant (TTP), effective tillers per plant (ETP), fertile spikelets per panicle (FSP), sterile spikelets per panicle (SSP), total spikelets per panicle (TSP), spikelet fertility % (SF%), hundred seed weight (HSW), grain yield per plant (GYP), hulling % (Hul%), milling % (Mil%), paddy length (PadL), paddy breadth (PadB), brown rice length (BRL), brown rice breadth (BRB), kernel length (KL), kernel breadth (KB), kernel length breadth ratio (KLBR), cooked rice length (CRL), cooked rice width (CRW), elongation ratio (ER), alkali spreading value (ASV), gel consistency (GC), amylase content (AC%) and head rice recovery % (HRR%) showed normal distribution patterns over the three seasons (Figure 2a–c and Supplementary Table S4), indicating that these traits have very less or negligible deviations in their mean values over the three generations. Further, this suggested that these traits might have high heritability and lesser influence from environmental factors.

### 2.4. Improvement in Days to 50% Flowering, Plant Height (cm) and Grain Yield per Plant (g) of the Rice Mutants over the Corresponding Parent

**Figure 3:**
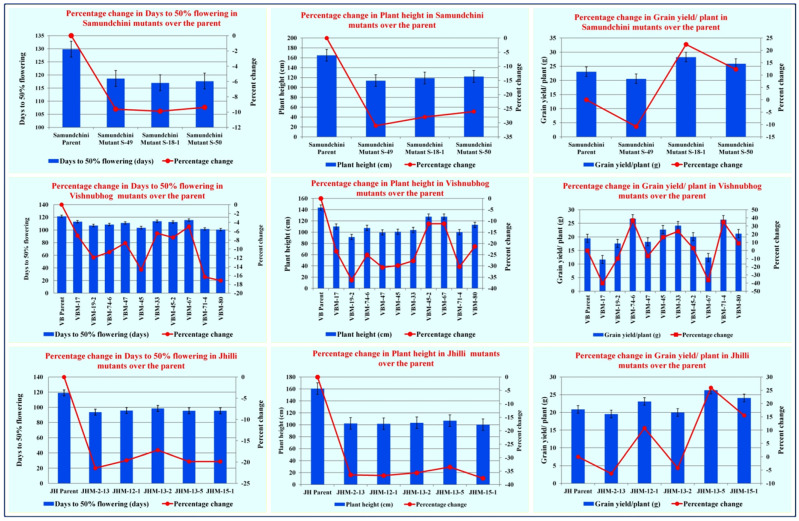
Percentage change in plant height, maturity duration and grain yield of mutants over the corresponding parents.

**Figure 4:**
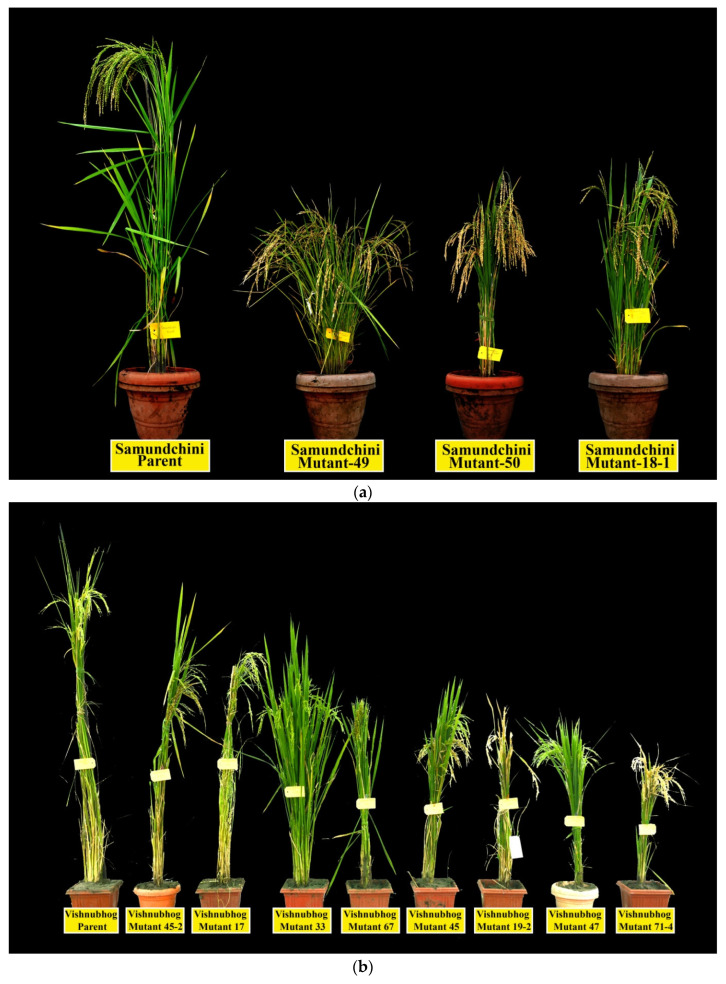

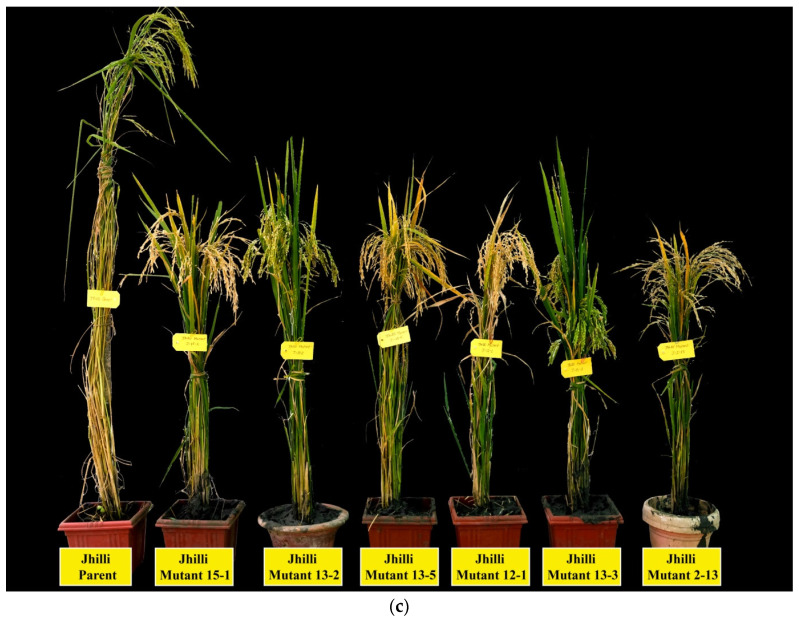
Comparative view of (**a**) Samundchini mutants, (**b**) Vishnubhog mutants and (**c**) Jhilli mutants with their corresponding parent.

In the present study, all the mutants of the three landraces showed a reduction in days to 50% flowering and plant height as compared to their parents in all the three generations (M4, M5 and M6 generations) (Table 2 and Figure 3). The average percent reduction in plant height in mutants of Samundchini, Vishnubhog and Jhilli was 28.35%, 24.74% and 35.96% respectively, whereas, for days to 50% flowering, 9.62%, 10.52% and 19.58% reduction was observed in Samundchini, Vishnubhog and Jhilli mutants, respectively. A remarkable reduction in DFF was recorded in Jhilli Mutant J-2-13 (21.40%), whereas a negligible reduction was observed in Vishnubhog mutant V-67 (4.94%). Similarly, the maximum reduction in plant height was observed in Jhilli Mutant J-15-1 (37.65%), whereas a minimum reduction was observed in Vishnubhog Mutant V-45-2 (11.28%) and Vishnubhog Mutant V-67 (11.28%). Comparative photographs of the mutants of Samundchini, Vishnubhog and Jhilli with their corresponding parents are given in Figure 4a–c, respectively.

Considering the grain yield/plant, two mutants of Samundchini: namely, Samundchini Mutant S-18-1 and Samundchini Mutant S-50, showed 22.45% and 12.32% increment in grain yield/plant over the parent. While comparing these mutants with the check varieties, it was observed that Samundchini Mutant S-18-1 had a 51.42% and 36.44% while Samundchini Mutant S-50 had a 38.94% and 25.19% higher yield over the check varieties Dubraj selection-1 and Vishnubhog selection-1, respectively, over the means of the three seasons (Table 2 and Figure 4a). However, Samundchini Mutant S-49 surpassed the check Dubraj Selection-1 with a 10.28% yield advantage but not the Vishnubhog Selection-1.

**Figure 5:**
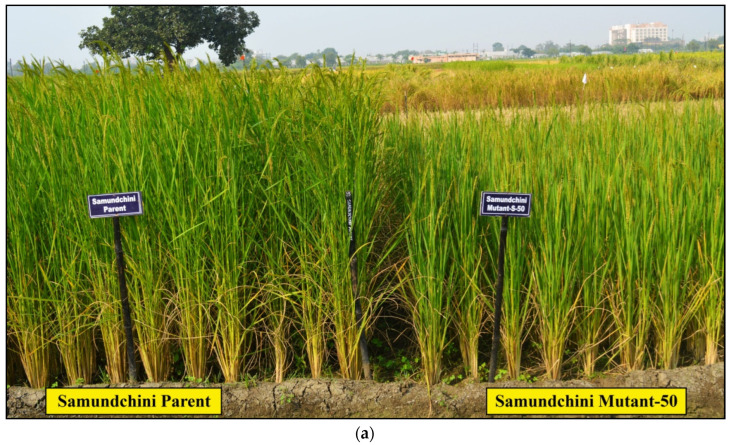

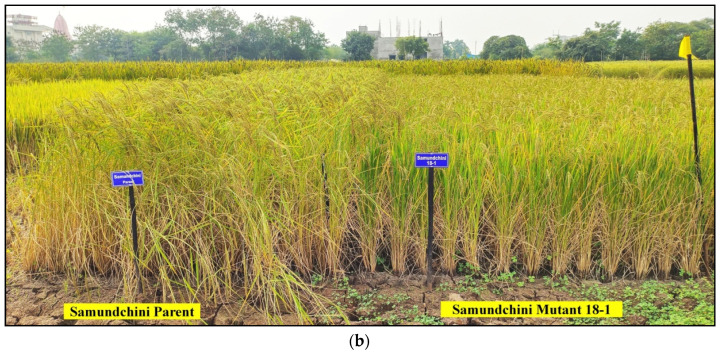
Comparative field views of the promising (**a**) Samundchini Mutant S-50 and (**b**) Samundchini Mutant S-18-1 with the Samundchini parent.

Samundchini Mutant S-49 showed a reduced yield, because it was a high tillering grassy type of mutant with a fine grain type, low test weight and low spikelet fertility compared to the parent. Moreover, the two prominent mutants showed improved plant canopy, high tillering, reduced plant height and earliness as compared to the parent. The better yield performance of the mutants showed a significant achievement towards the varietal development route. Based on the good performance of Samundchini Mutant S-50 and Samundchini Mutant S-18-1, they have been nominated for multilocation trials at the state and national level for the years 2021 and 2022. Comparative field views of promising Samundchini Mutant S-50 and Samundchini Mutant S-18-1 are given in Figure 5a,b, respectively.

**Figure 6:**
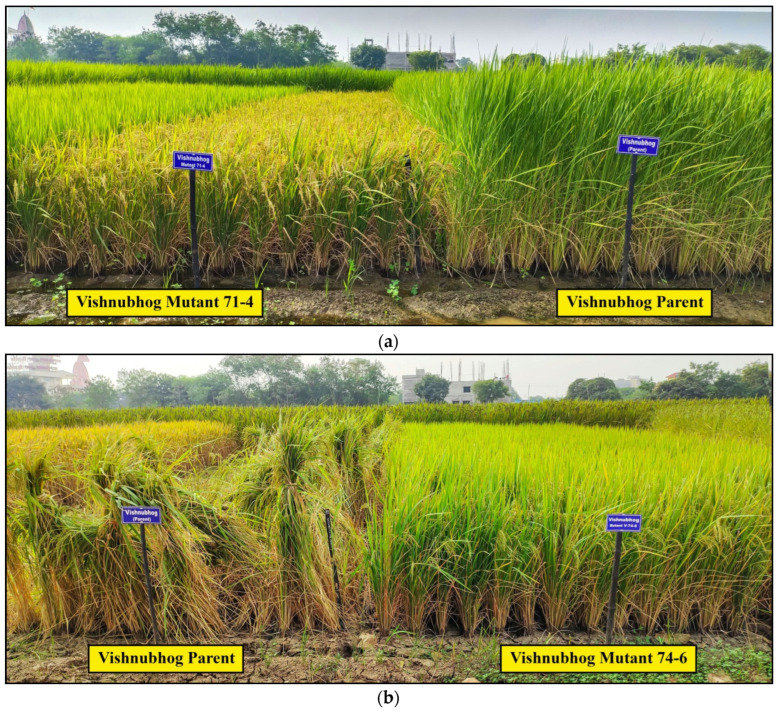

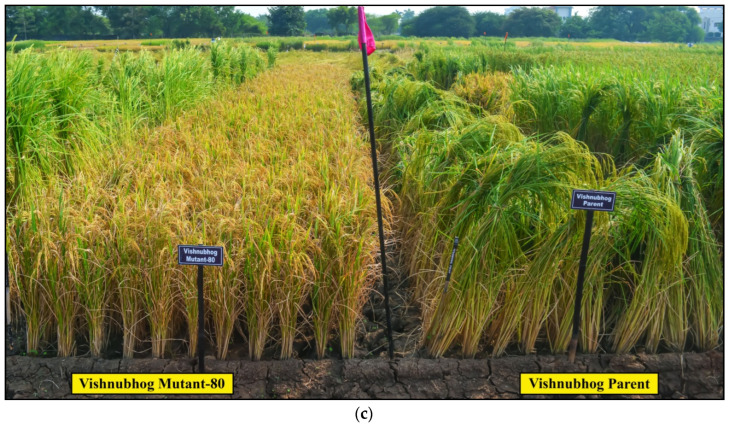
Comparative field views of promising (**a**) Vishnubhog Mutant 71-1, (**b**) Vishnubhog Mutant 74-6 and (**c**) Vishnubhog Mutant 80 with the Vishnubhog parent.

In Vishnubhog, six out of ten mutants showed a yield advantage over the parent, as depicted by the pooled mean of the grain yield/plant over three seasons (Table 2 and Figure 3 and Figure 4b). The highest percent increase in grain yield was observed in Vishnubhog Mutant V-74-6 (36.87%), followed by Vishnubhog Mutant V-71-4 (34.93%), Vishnubhog Mutant V-33 (23.75%), Vishnubhog Mutant V-45 (16.46%) and Vishnubhog Mutant V-80 (8.89%). Among these high-yielding mutants, Vishnubhog Mutant V-80 has highly aromatic grains with slightly fine grains compared to the parent.Furthermore, while comparing with check varieties Dubraj Selection-1 and Vishnubhog Selection-1, Vishnubhog Mutant V-74-6 showed the highest yield advantage (43.12% and 28.96%), followed by Vishnubhog Mutant V-71-4 (41.08% and 27.12%), Vishnubhog Mutant V-33 (29.41% and 9.70%), Vishnubhog Mutant V-45 (21.75% and 9.70%) and Vishnubhog Mutant V-80 (13.87% and 2.61%) over the check varieties Dubraj Selection-1 and Vishnubhog Selection-1, respectively, over the mean of three seasons (Table 2). Based on the good mean performance of these mutants, Vishnubhog Mutant V-74-6, Vishnubhog Mutant V-71-4 and Vishnubhog Mutant V-80 have been nominated for multilocation trials at the state and national levels for the years 2021 and 2022. Comparative field views of promising Vishnubhog Mutant 71-4, Vishnubhog Mutant 74-6 and Vishnubhog Mutant-80 are given in Figure 6a–c.

**Figure 7:**
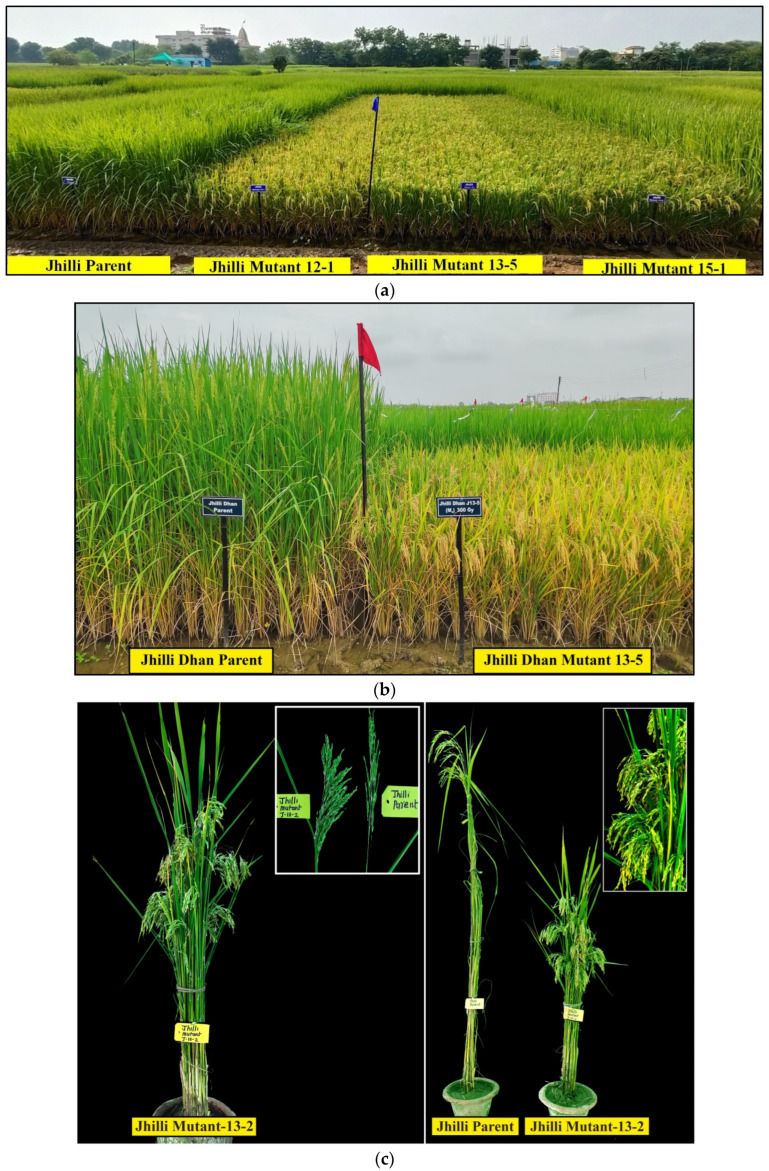
(**a**) Comparative field view of promising Jhilli Mutant 12-1, Jhilli Mutant 13-5 and Jhilli Mutant 15-1 with Jhilli parent. (**b**) Comparative field view of promising Jhilli Mutant 13-5 with the Jhilli parent. (**c**) Representation of Jhilli Mutant 13-2 having clustered grains.

In Jhilli, three out of five mutants showed a higher yield as compared to their parent. The highest yield advantage was observed in Jhilli Mutant J-13-5 (25.96%), followed by Jhilli Mutant J-15-1 (15.51%) and Jhilli Mutant J-12-1 (10.78%) (Table 2 and Figure 3 and Figure 4c). Moreover, while comparing with the check varieties Dubraj Selection-1 and Vishnubhog Selection-1, Jhilli Mutant J-13-5 showed the highest yield advantage (40.71% and 26.79%), followed by Jhilli Mutant J-15-1 (29.03% and 16.26%) and Jhilli Mutant J-12-1 (23.73% and 11.49%), respectively, over the mean of three seasons (Table 2). These high-yielding mutants with reduced height and maturity durations have been nominated for multilocation trials at the state level for the years 2021 and 2022. The field view of some promising mutants Jhilli Dhan are given in Figure 7a–c. Interestingly, Jhilli Mutant 13-2 has clustered grains as compared to the parent (Figure 7c).

It was observed that the trait plant height was altered significantly compared with the days to 50% flowering in all the mutant lines. Plant height is an important character in rice breeding, because it is strongly associated with the effective utilisation of plant assimilation to improve the plant yield. Genotypes with high production are characterised by short stems, which makes the division of assimilation very effective [10]. A similar study was performed by Roy et al. [20], who reported a rice mutant with a high yield (>89% increase in yield) over the control cultivar. Sharma et al. [19] reported 3–49.9% yield increment in Dubraj mutants. Kato et al. [22] reported 1.2–22.5% higher yield than their original parent. In the outcomes of the present study, we assume that the higher yields of the selected mutants could have been because of the directed selection pressure towards the yield-attributing traits, which showed pleiotropic effects ensuring improvement in the physiological process of the accumulation of photo assimilates, resulting in a higher yield of mutants.

### 2.5. Analysis of Variance (ANOVA) Based on Randomised Complete Block Design (RCBD)

ANOVA based on RCBD divided the total variations into three components, i.e., variations due to genotypes, environmental conditions and interactions between genotypes and environmental conditions. The results revealed that the mean sum of squares due to the genotypes were highly significant for all the characters under consideration, indicating that ample variability exists in all the genotypes for all the traits (Table 3). Interestingly, few previous studies also reported to have significant variations among the genotypes for yield-attributing traits and grain quality traits, which corroborated the outcomes of the present study [23,24,25,26]. Moreover, the mean sum of squares due to the different environmental conditions were also significant for most of the characters taken in the study, except for Pad L, Pad W, KLBR and CRW. Moreover, interactions between genotypes and the environment were significant for most of the traits, viz., DFF, PH, PL, FLL, FLW, TTP, ETP, FSP, SSP, SF%, HSW, GYP, Hul%, Mil%, Pad L, BRB, KLBR, CRL, CRW, ER, ASV, GC and AC, except for Pad L, Pad W and HRR (%) (Table 3). It showed a significant effect of the interactions on the treatment performance; hence, the factors considered to split the variance into different components were appropriate. In addition, different environmental conditions possessing different weather parameters have put significant effects on the expression of traits. Interestingly, Konate et al. [27], Tiwari et al. [28] and Sahu et al. [29] also observed significant interactions between genotype and environment for agro-morphological and grain quality traits in rice in their studies.

### 2.6. Genetic Variability Parameters for Agro-Morphological and Grain Quality Traits

The study of the coefficients of variation indicated that the estimates of the phenotypic coefficient of variation (PCV) were slightly higher than the corresponding genotypic coefficient of variation (GCV) estimates for all traits during all three generations or seasons, indicating that the phenotypic variation was largely accounted for by the genotype, with a negligible influence of the extraneous factors, and therefore, the selection for such traits on the basis of phenotype could be rewarding (Table 4). Moreover, the highest values of PCV and GCV were recorded for the total number of tillers/plants in all three generations; their values also showed increases as the generations advanced. Interestingly, several traits, viz., FLW, TTP, ETP, HSW, GYP, KL, KLBR, ASV and GC, showed high GCV coupled with high PCV consistently in all three generations, indicating the presence of abundant variability and direct selection would be rewarding for the improvement of those traits (Table 4). Furthermore, the elongation ratio, kernel length and breadth ratio and kernel length after cooking showed high/moderate GCV coupled with high PCV indicating the existence of comparatively moderate variability for these traits, which could be exploited for improvement through selection in advanced generations. Interestingly, the researchers, viz., Chaudhari et al. [30], Nayak et al. [31], Chakraborty et al. [32], Vanaja and Babu [33], Sanjukta et al. [34], Veerabadhiran et al. [35], Prashanth et al. [25] and Adewusi et al. [26] also observed high to moderate GCV with high PCV for the agro-morphological traits and grain quality traits in rice under their studies, which supported the results of the current study.

The estimates of heritability act as the predictive index in expressing the reliability of the phenotypic value. Therefore, high heritability helps in the effective selection for a particular character. In the M4, M5 and M6 generations, the highest broad sense heritability was exhibited for plant height (98.97%), milled rice length (98.96%) and plant height (99.21%), respectively. In all three generations, most of the traits showed high heritability, except for hulling (%), milling (%) and HRR (%) in the Kharif season 2019 (M4 generation) and fertile spikelets/panicle and HRR (%) in the Rabi season 2020–21 (M5 generation), which showed moderate heritability (%) (Table 4). Furthermore, high to moderate heritability values indicated that the characters under study less influenced by the environment in their expression and simple or direct selection based on the phenotype would ultimately improve the genetic backgrounds of these traits [36,37,38,39].

The estimates of genetic advance (GA) as a percent of the mean provides more reliable information regarding the effectiveness of the selection for improving the traits. Most of the traits studied exhibited a high estimate of genetic advance as a percent of the mean in all three generations (M4, M5 and M6), except for spikelet fertility (%), dehusked rice width, milled rice width, cooked rice width and amylose content, which showed moderate values and hulling (%), milling (%) and HRR (%), which showed lower values (Table 4).

Traits, which showed high, as well as moderate GA, as a percent mean, were governed by additive gene action, and direct selections for such traits might be rewarding [39,40,41], whereas the three traits hulling (%), milling (%) and HRR (%) showed low GA as a percent mean in all three generations, indicating the influence of nonadditive gene action and heterosis breeding may be rewarding for the improvement of such traits.

Heritability, along with the genetic advance, is the important selection parameters for predicting the genetic gain under selection. Interestingly, the traits, viz., days to 50% flowering, plant height, panicle length, flag leaf length, flag leaf width, total tillers/plant, effective tillers/plant, fertile spikelets/panicle, 100-seed weight, grain yield/plant, paddy length, paddy width, dehusked rice length, milled rice length, kernel L/B ratio, cooked rice length, elongation ratio, alkali spreading value and gel consistency showed consistently high heritability coupled with high genetic advance as a percent mean over the three seasons, indicating the lesser influence of the environment in their expression and prevalence of additive gene action in their inheritance, are hence amenable for simple and direct selection for their improvement [23,24,25,39].

Moreover, high heritability coupled with moderate GA as a percent mean was observed for spikelet fertility%, dehusked rice width, milled rice width, cooked rice width and amylose content, suggesting the role of both additive and nonadditive gene actions in their inheritance, and selection after the hybridisation would be more appropriate rather than direct selection [42]. Furthermore, none of the traits in all the three generations were found to have low values of heritability with low genetic advance indicating traits taken under study are less influenced by environment and selection can be performed.

### 2.7. Association Analysis Based on Agro-Morphological and Grain Quality Traits in M4, M5 and M6 Generations

**Figure 8:**
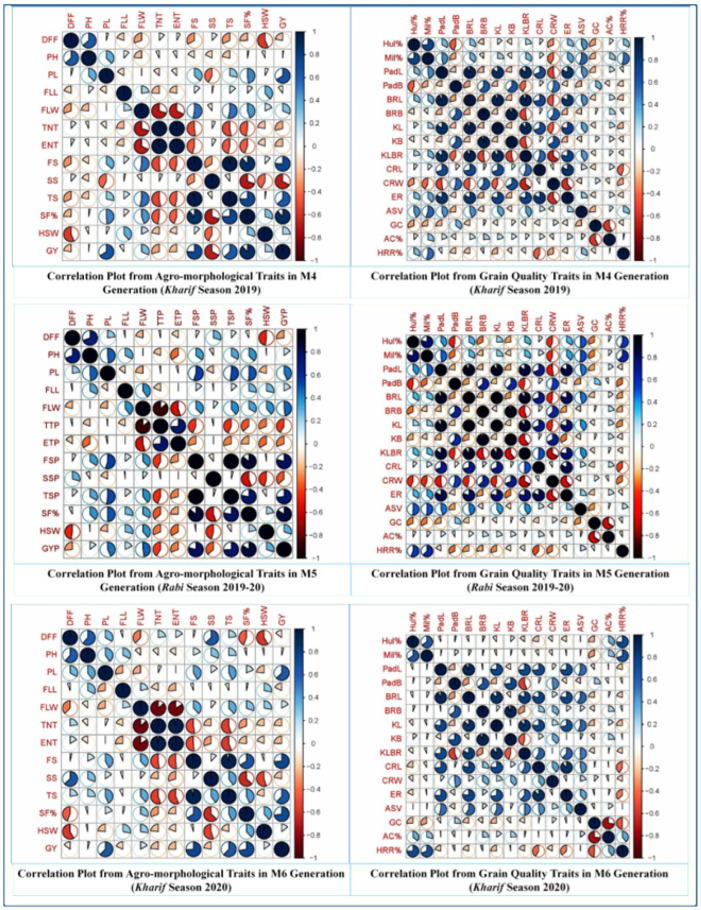
Correlation plots for the agro-morphological and grain quality traits during all three seasons.

The association analysis gives an idea about the relationships among the various characters and determines the component characters on which selection can be based for genetic improvement in the grain yield and quality traits. In the present study, the correlation coefficient for grain yield per plant showed positive and significant associations with the panicle length, flag leaf width, fertile spikelets/panicle, total spikelets/panicle, spikelet fertility (%) and 100-seed weight, whereas it showed significant negative correlation with plant height and sterile spikelets/panicle in all three generations (Supplementary Table S5 and Figure 8). Moreover, in the case of grain quality parameters, the correlation coefficient for the head rice recovery percent (HRR%) showed a positive and significant association with hulling (%) and milling (%), whereas it showed a significant negative correlation with the cooked rice length in all three generations. Furthermore, hulling (%) and milling (%) were also positive and significantly associated with each other during all three generations (Supplementary Table S6 and Figure 8).

The correlation coefficients, along with path coefficients, together provide more reliable information, which can be effectively predicted in the crop improvement program. If the correlation between causal factor and direct effects is more or less of equal magnitude, it explains the true and perfect relationship between the traits, and hence, direct selection through these traits will be rewarding. However, if the correlation coefficient is positive and the direct effects are negative or negligible, the indirect causal factors are to be considered in simultaneous selection.

A path coefficient study was carried out separately by considering the grain yield/plant and head rice recovery percent (HRR%) as the dependent variable for morphological and grain quality traits, respectively, and the rest of the characters were considered as independent variables in all three generations. The correlation coefficients were partitioned into direct and indirect effects in the path coefficient analysis, which are presented in Table 5 and Table 6 for the grain yield-attributing traits and grain quality traits, respectively. In all three generations or seasons, the panicle length, total tillers/plant, fertile spikelets/panicle, total spikelets/panicle, spikelet fertility (%) and 100-seed weight were the common traits showing high direct effects and significant positive correlations with the grain yield/plant. In addition, the plant height and sterile spikelets/plant showed significant negative correlations and negative direct effects with the grain yield per plant.

Moreover, based on the direct and indirect effects recorded for the grain quality traits, hulling (%) and milling (%) were the common traits showing high direct effects and significant positive correlations with HRR (%), whereas milled rice length and cooked rice length showed a high direct effect, along with the significant negative correlation for HRR (%) during all three generations. Traits showing a positive correlation and high direct effect with the dependent variable indicated a true relationship between them, and directly selection favouring those traits would be very effective for improving the yield potential and head rice recovery (%) of the rice genotypes [23,25,43,44], while traits showing negative correlations and negative direct effects would not be good for improving the dependent variable [45,46,47,48]. The residual effects were very low in all three generations, indicating that the characters included under study were sufficient for improving the dependent variable through selection.

### 2.8. SSR Marker-Based Genomic Similarity and Genetic Diversity Study in Rice Mutants and Parents

#### 2.8.1. SSR Marker Profile and Informativeness Used for Genotyping of Rice Genotypes

A total of 46 SSR markers randomly dispersed across the 12 chromosomes of rice were used to evaluate the genomic similarity of mutants and parents and genetic diversity of the 24 rice genotypes (Table 7). The number of alleles per loci varied from 1 (RM452) to 5 (RM55), with an average of 2.56 alleles per locus. Overall, the polymorphic information content (PIC) value ranged from 0 (RM484) to 0.68 (RM408), with an average of 0.37. The expected heterozygosity or gene diversity (He) computed according to Nei [49] varied from zero to 0.73 (RM408), with an average of 0.43. Truong et al. [44] also reported a range of PIC values from 0.09 to 0.79, with an average of 0.47, in rice mutants through SSR markers, which is in agreement with the present study. The major allele (most common) frequency ranged from 0.37 (RM489) to 1 (RM552), with an average of 0.66. In this study, the genetic diversity analysis revealed a mean for the allele number (2.56), major allele frequency (0.66), number of genotypes (2.59), gene diversity of (0.43) and PIC value of 0.37. These values are similar to those previously reported in studies on rice by Garris et al. [50], Liakat-Ali et al. [51] and Jamil et al. [52].

#### 2.8.2. SSR Marker-Based Genome Similarity Study in Rice Mutants and Their Corresponding Parents

The genome similarity study between mutants and their corresponding parents revealed that 73, 53 and 60 alleles were amplified for Vishnubhog, Samundchini and Jhilli mutants, respectively. Among the Vishnubhog mutants, the highest similarity was depicted by Vishnubhog Mutant-71-4 (95.65%) and Vishnubhog Mutant-74-6 (95.65%) with Vishnubhog parent, followed by Vishnubhog Mutant-19-2 (93.40%) and others (Table 8).

In Samundchini, Samundchini Mutant S-49 had the highest similarity (95.65%) with the parent, followed by Samundchini Mutant S-50 (91.30%) and Samundchini Mutant S-18-1(89.13%). In Jhilli, the highest similarity percent was observed in Jhilli Mutant J-2-13 (95.65%), followed by Jhilli Mutant J-13-5 (91.30%), Jhilli Mutant J-15-1 (89.13%) and Jhilli mutant J-12-1 (80.43%), with the parent. Similarity shows the trueness of mutants with their corresponding parent, whereas dissimilarity shows the effect of mutagenesis at different SSR loci, which generated different alleles in the mutants with respect to their parents.

Similarly, Potupureddi et al. [53] and Suneel et al. [54] also obtained 85.0–98.3% genomic similarity between the rice mutants and parents using SSR markers. Moreover, Andrew-Peter et al. [55] also reported the minimum dissimilarity and maximum similarity values between mutants and parents in rice. On concluding, all the mutants taken for the study showed much genomic similarity (percent), which showed that all these mutants were the same as that of the parent in most of the traits/genes, while the differences present for only one or few characters in the mutants are shown by the appearance of some other alleles.

#### 2.8.3. SSR Marker-Based Genetic Diversity Study in Rice Genotypes

Molecular markers also facilitate to understand the level of genetic diversity that exists among the genotypes, which can be exploited in rice breeding programs. The unweighted pair group method with arithmetic mean (UPGMA) algorithm and Gower distance-based cluster analysis divided the total 24 rice genotypes into three main clusters: cluster-A, cluster-B and cluster-C at a Gower distance of 0.32. The cluster diagram shows that the Gower Distance ranges from 0.04 to 0.38. A lower Gower distance shows a higher similarity between the genotypes and vice versa.

**Figure 9:**
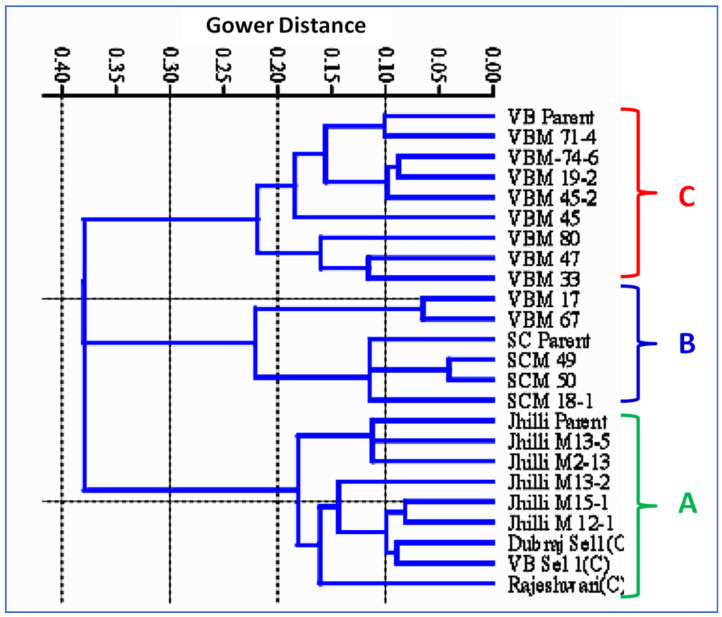
SSR marker-based cluster diagram (dendrogram) showing the similarities and dissimilarities between the mutants and parents.

The cluster diagram revealed that the mutants were grouped with their respective parents and went into separate clusters. Accordingly, three major clusters were formed for Samundchini, Vishnubhog and Jhilli (Figure 9). All the mutants of Jhilli, along with the parent and three checks, were grouped into cluster-A. Similarly, cluster-B has six genotypes, viz., the parent and mutants of Samundchini, along with two mutants of Vishnubhog. Likewise, cluster-C contains nine genotypes, including the Vishnubhog parent and its eight mutants. Two mutants of Vishnubhog fall under cluster-B with the Samundchini mutants; this might be because the grain type of Vishnubhog Mutant V-17 changed from short bold to medium slender after mutagenesis, which is similar to the grain type of Samundchini. Additionally, the arrangement of grains in the panicles of Vishnubhog Mutant V-67 changed to a clustered type of arrangement with a reduced grain length, which made it fall into cluster-B. The three checks included under the study were grouped with mutants of Jhilli, which might be because of the long grain type as that of Jhilli. The highest similarity was shown between Samundchini Mutant S-49 and the Samundchini parent, as they showed the lowest Gower distance. This study shows that almost all the mutants of the three parental genotypes taken under study were true to type to their respective parents at the molecular level. 

**Figure 10:**
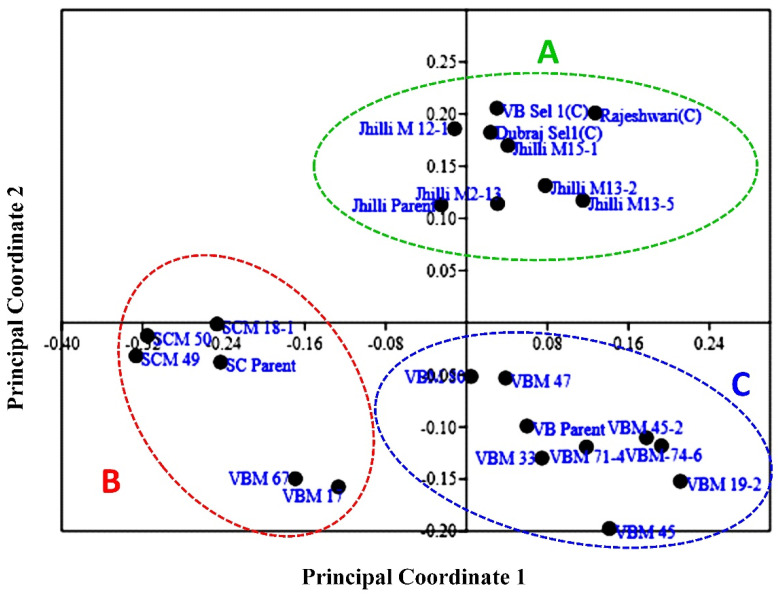
PCA scatter diagram showing the grouping pattern of rice mutants and parents based on SSR markers.

**Figure 11:**
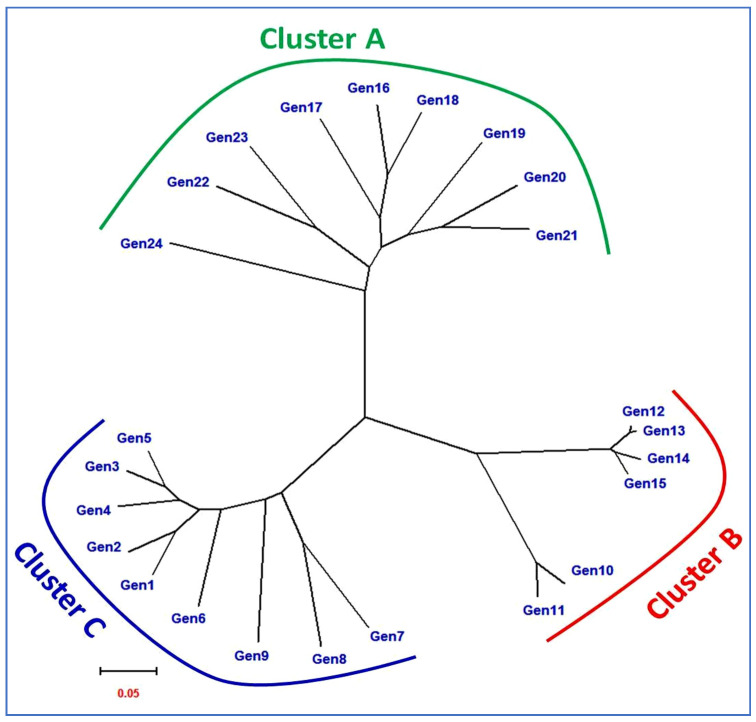
Neighbour joining tree showing the grouping pattern of rice genotypes.

The genome similarity of the mutants with their parents supports the fact that most of the traits of the mutants were same as that of their parents, except one or a few that were a prerequisite for a mutant to come into being and could precede forward towards varietal development. Further, Oladasu et al. [10] performed a cluster analysis on rice mutants and parents using ISSR markers and reported that the genetic similarity coefficient ranged from 0.17 to 0.63, which is in agreement with the genetic Gower distance (0.04–0.38) obtained in the present study. Furthermore, Kumar and Bhagwat [56], Oladasu et al. [10], Truong et al. [44], Suneel et al. [54], Potupureddi et al. [53] and Andrew-Peter et al. [55] also performed UPGMA-based cluster analyses on rice mutants and parents and reported similar findings as reported in the current study. Furthermore, the principal component analysis (PCA)-based scatter plot prepared over the principal coordinate 1 (PC1) and principal coordinate 2 (PC2) clearly demonstrated the patterns of the three major groups’ (A, B and C) differentiations (Figure 10). The three groups majorly comprised of the three rice landraces within the study, along with their mutants. Moreover, three checks were grouped with the Jhilli mutants in Group-A. The scatter diagram revealed that all the long grain rice genotypes comprising the mutants of Jhilli, Jhilli parent and three checks were grouped into group-A, whereas all medium slender grain genotypes consisting of Samundchini mutants, along with the parents and two mutants of Vishnubhog (Vishnubhog mutant-V-17 and Vishnubhog mutant-V-67), were grouped into group-B along the PC2 axis. Moreover, short and medium bold mutants of Vishnubhog, along with the control, were grouped into group-C. Additionally, the neighbour joining tree showed the clear formation of the three subgroups comprising the genotypes falling into the same subgroups formed as that of the PCA (Figure 11). It depicted that the mutants of the respective parents were less genetically diverse or showed more similarities among themselves, as well as with their parents. Although some level of genetic diversity was shown among the mutants of the same genotype, depicting the discrete identity of every mutant. The additional formation of the groups at different ordinates showed that the genotypes falling into different groups were genetically diverse from the genotypes of the other such groups. These findings were similar with the results of the similarity coefficient analysis and clustering based on the UPGMA algorithm and Gower distance. Both analyses showed grouping/clustering of similar related genotypes and helped us to identify diverse parents that could be further used in the hybridisation program. 

The overall results obtained from molecular data indicate that irradiation might introduce significant levels of genetic and morphological diversities into mutant lines relative to what is inherent from the parent. Additionally, on correlating the clustering patterns obtained through quantitative traits and molecular data, we obtained similar groupings of most of the genotypes in their respective clusters, which showed an authenticity of genetic similarity resulting from analyses of both quantitative traits and molecular data. Genetic diversity was evident through the used quantitative traits, and it was confirmed at the molecular level [57,58]. This was attested to by the results of the correlations between molecular and quantitative trait parameters in our study. Additionally, our results also showed that irradiation could generate a considerable amount of genetic variability and provide new avenues for crop improvement and diversification. This crop improvement might also be successful in the development of mutant lines with desirable traits that are absent in the parent [59].

## 3. Materials and Methods

### 3.1. Experimental Materials

Three traditional rice landraces, viz., Vishnubhog, Samundchini and Jhilli, were taken as base materials for the present study. These landraces are admired in specific districts of Chhattisgarh (C.G.), India, i.e., Bilaspur, Sarguja and Mahasamund, for Samundchini, Vishnubhog and Jhilli, respectively. Seeds of these landraces were procured from the R.H. Richharia Rice Germplasm Division of the Department of Genetics and Plant Breeding, Indira Gandhi Krishi Vishwavidyalaya, Raipur (C.G.), India. The specific features and lacunas of these landraces are given in Supplementary Table S7. In addition, 24 rice genotypes, including parental genotypes, their respective mutants and three checks, viz., Rajeshwari, Dubraj Selection-1 and Vishnubhog selection-1, were used in this study in advanced generations, viz., the M4, M5 and M6 generations (Table 9).

### 3.2. Gamma Ray Irradiation Facility

Around 2000 dried, pure and healthy seeds of Samundchini, Vishnubhog and Jhilli were irradiated with a dose of 300 Gy gamma rays using a Cobalt-60 isotope-based gamma source (GC-5000, Board of Radiation Isotope Technology, Mumbai India) with a dose rate of-37 Gy/min at Nuclear Agriculture and Biotechnology Division (NA&BTD), Bhabha Atomic Research Centre, Mumbai, India. In order to optimise the lethal dose 50 (LD50) and growth reduction 50 (GR50) doses, seeds were treated with different doses of gamma rays, and the optimum dose was calculated based on the germination (%), shoot length, root length, seedling length and vigour index. The results regarding the dose optimisation study have been already published [60].

### 3.3. Experimental Site, Setting of the Experiment and Agronomic Practices Adopted

The experiment was carried out at the research field of the Department of Genetics and Plant Breeding, Indira Gandhi Krishi Vishwavidyalaya (IGKV), Raipur-492012 (C.G.), India. Raipur, the capital of Chhattisgarh State, India, lies at 21°16′ N latitude and 81°36′ S longitude, with an altitude of 289.60 m above the mean sea level. The average rainfall of the region is 1200–1400 mm per annum, most of which is received during June–September, with occasional light showers during the winter and summer seasons.

The spacing of 20 cm between rows and 15 cm between plants was maintained. Recommended doses of the fertilisers were applied, and the required plant protection measures were taken at the different stages of crop growth. All the required agronomic and cultural practices were adopted as per the standard methodology throughout the crop season.

### 3.4. Handling of M1 Population

The treated seeds of all three rice landraces were sown separately as the M1 population during the dry (Rabi) season 2017–18 at the experimental site. The mother panicle from each M1 plant was harvested and kept separately to be sown in the next generation constituting the M2 population.

### 3.5. Handling of M2 Population and Selection of Desirable Mutants

Harvested M1 seeds of each landrace were grown as the M2 population in the experimental field during the wet (Kharif) season 2018 by following the panicle to row method. A total of 13,712, 12,306 and 11,744 plants of Samundchini, Vishnubhog and Jhilli, respectively, were maintained as the M2 population. Screening/selection for all possible types of morphological changes (putative chlorophyll and viable mutants) that deviated from the untreated (control) plants was done [61]. The total selected putative mutants were tagged and harvested separately.

### 3.6. Handling of M3 Population

Seeds from all the selected mutants were sown as the M3 population during the dry (Rabi) season 2018–19 by adopting the plant to row method. Chlorophyll mutants were not included for further generation advancement study. Trait expressions of the putative mutants were confirmed through selection in this generation, whereas those that deviated from the traits under selection and with poor performance were discarded.

### 3.7. Characterisation and Evaluation of Rice Genotypes in M4, M5 and M6 Generations and Experimental Layout

Eighteen mutants, which included three mutants of Samundchini, ten mutants of Vishnubhog and five mutants of Jhilli, were selected from the M3 generation for study in further generations. A total of 24 genotypes (18 mutants, their corresponding three parents and three checks) were grown in replicated yield trials by following Randomized Complete Block Design (RCBD) with two replications during the wet (Kharif) season 2019 (M4 generation), dry (Rabi) season 2019–20 (M5 generation) and wet (Kharif) season 2020 (M6 generation). The layout plan adopted for the RCBD during all three seasons is given in Supplementary Figure S1.

### 3.8. Observations Recorded for Yield-Attributing Traits and Physicochemical Grain Quality Traits in M4, M5 and M6 Generations

All 24 rice genotypes were evaluated for thirteen yield-attributing traits, viz., days to 50% flowering (DFF), plant height (PH), panicle length (PL), flag leaf length (FLL), flag leaf width (FLW), total number of tillers/plant (TTP), effective number of tillers/plant (ETP), fertile spikelets/panicle (FSP), sterile spikelets/panicle (SSP), total spikelets/panicle (TSP), spikelet fertility%, (SF%) 100-seed weight (HSW) and grain yield/plant (GYP), at the appropriate plant stage by following the Standard Evaluation System (SES)-2013 of International Rice Research Institute [62]. Additionally, sixteen grain quality parameters, viz., hulling% (Hul%), milling% (Mil%), Head Rice Recovery% (HRR%), paddy length (PadL), paddy width (PadW), dehusked rice length (BRL), dehusked rice width (BRB), milled rice length (KL), milled rice width (KB), kernel L/B ratio (KLBR), cooked rice length (CRL), cooked rice width (CRW), elongation ratio (ER), alkali spreading value (ASV), gel consistency (GC) and amylose content (AC), were recorded in all three generations at the Crop Quality Laboratory of Department of Genetics and Plant Breeding, R.H. Richharia Rice Research Laboratory, IGKV, Raipur (CG), India, by following the standard protocol developed by the ICAR-Indian Institute of Rice Research (IIRR), Hyderabad [63].

### 3.9. Molecular Profiling of the Rice Mutants, Parents and Checks with the Help of SSR (Simple Sequence Repeat) Markers

A SSR marker-based genotyping and genome similarity study was performed for eighteen rice mutants, along with their parents and three checks. A set of 50 SSR markers was taken from the panel of 50 standard SSR markers of the IRGSP Generation Challenge Program, as given in the Gramene marker database (https://archive.gramene.org/markers/microsat/50_ssr.html, accessed on 2 April 2020). However, 46 markers were clearly amplified; therefore, those 46 were used for further analysis. The details of these markers are given in the Results section.

### 3.10. Bio-Statistical Analysis Performed with the Data Obtained in M4, M5 and M6 Generations

The observations recorded on various yield-attributing traits and grain quality traits were subjected to statistical analysis by using statistical software packages. Pooled analysis of variance (RCBD); genetic variability parameters; correlation and path coefficients were estimated with the help of the SAS-based OPSTAT online tool (http://14.139.232.166/opstat/, accessed on 5 March 2021). Correlation graphs and frequency distribution-based histograms were prepared by R-studio. Shapiro–Wilk W base normal distribution of the traits was performed by PAST v3.15 software [64]. Marker utility information and genetic diversity parameters for each marker were estimated using the program POWERMARKER version 3.25 [65]. A genome similarity study was performed based on the similar alleles amplified in both mutant and parent. The number of similar alleles present in each mutant when compared with the respective parent, on the basis of which a similarity percent of mutants with respect to parent was computed through MS Excel. The allele size data of all 46 SSR markers in all the rice genotypes were subjected to a cluster analysis by following the UPGMA (unweighted pair group method with arithmetic mean) algorithm and Gower genetic distance with the help of PAST v3.15 software [64].

## 4. Conclusions

Despite possessing huge potential for crop improvement, rice landraces are endured with one or few major problems, viz., poor yield potential, tall plant stature, late maturity duration, grain shattering, uneven maturity of grains, spreading plant type, etc., which restrict their cultivation in farmers’ fields. Among several approaches available for mitigating the limitations of these rice germplasms, radiation-induced mutation breeding is a relatively quicker and efficient and more viable method for the improvement of one or a few major undesirable traits. Therefore, the present study was conducted with the aim of genetic improvement of the tall, late maturing and poor-yielding rice landraces through gamma ray-induced mutagenesis. The major outcome of this study was that the mutants screened in all three genotypes had significantly reduced plant height, reduced maturity duration and better yield performance as compared to their corresponding parents. Further, the trueness of the mutants was evaluated through a SSR marker-based genomic similarity study, which revealed a more than 90% similarity in most of the mutants with their parents, while a minor dissimilarity was due to alterations in one or a few characters. This study will open up many avenues for rice breeders in planning, execution and delivering good products through radiation-induced mutation breeding.

## 5. Variety

Vishnubhog Mutant V-80 developed through this work has been released by the State Variety Release Committee, Chhattisgarh, India, and notified by the Government of India for its commercial cultivation in Chhattisgarh State, India, on 3 January 2022, with gazette notification no. SO 8(E). It has also been registered in the FAO/IAEA Mutant Variety Database (MVD) in 2022 (https://nucleus.iaea.org/sites/mvd/SitePages/Search.aspx?MVID=4940, accessed on 10 October 2022).

## Figures and Tables

**Table 1:** Details of true to type mutants and their special features as obtained in the M3 generation.

S. No.	Name of Mutants	Parental Lines	Features Based on Which They Have Been Selected and Advanced in Next Generation
1	Vishnubhog Mutant V-17	Vishnubhog	Very high tillering, semi dwarf, mid early maturity duration
2	Vishnubhog Mutant V-19-2	Vishnubhog	Semi dwarf, mid early maturity duration
3	Vishnubhog Mutant V-74-6	Vishnubhog	Semi dwarf, mid early maturity duration
4	Vishnubhog Mutant V-47	Vishnubhog	Semi dwarf, mid early maturity duration
5	Vishnubhog Mutant V-45	Vishnubhog	Semi dwarf, mid early maturity duration
6	Vishnubhog Mutant V-33	Vishnubhog	Semi dwarf, mid early maturity duration
7	Vishnubhog Mutant V-45-2	Vishnubhog	Semi dwarf, mid early maturity duration
8	Vishnubhog Mutant V-67	Vishnubhog	Semi dwarf, mid early maturity duration, clustered grain short panicle
9	Vishnubhog Mutant V-71-4	Vishnubhog	Semi dwarf, mid early maturity duration
10	Vishnubhog Mutant V-80	Vishnubhog	Intermediate type plant height, mid early maturity duration, fine grain type
11	Samundchini Mutant S-49	Samundchini	Very high tillering, intermediate type plant height
12	Samundchini Mutant S-18-1	Samundchini	Intermediate type plant height, early maturity
13	Samundchini Mutant S-50	Samundchini	Intermediate type plant height, early maturity
14	Jhilli Mutant J-2-13	Jhilli	Early, semi dwarf, panicles with clustered grains
15	Jhilli Mutant J-12-1	Jhilli	Early, semi dwarf
16	Jhilli Mutant J-13-2	Jhilli	Early, semi dwarf
17	Jhilli Mutant J-13-5	Jhilli	Early, semi dwarf
18	Jhilli Mutant J-15-1	Jhilli	Early, semi dwarf

**Table 2:** Percentage change in days to 50% flowering, plant height and grain yield/plant of mutants over the corresponding parents and checks.

List of Genotypes	Days to 50% Flowering (Days)	Percentage Change (%) over Parent	Plant Height (cm)	Percentage Change (%) over Parent	Grain Yield/Plant (g)	Percentage Change (%) over Parent	Percentage Change in Grain Yield (%) over Check 1 (Dubraj Selection-1)	Percentage Change in Grain Yield (%) over Check 2 Vishnubhog Selection-1 (%)
**Samundchini Parent**	**129.83**		**165.26**		**23.09**			
Samundchini Mutant S-49	118.67	−9.62	113.93	−31.06	20.59	−10.84	10.28	−0.63
Samundchini Mutant S-18-1	117.00	−9.88	119.05	−27.96	28.27	22.45	51.42	36.44
Samundchini Mutant S-50	117.67	−9.37	122.25	−26.03	25.94	12.32	38.94	25.19
**Vishnubhog Parent**	**121.50**		**143.69**		**19.52**			
Vishnubhog Mutant V-17	113.00	−7.00	109.95	−23.48	11.68	−40.19	−37.44	−43.63
Vishnubhog Mutant V-19-2	107.00	−11.93	91.49	−36.33	17.63	−9.70	−5.57	−14.91
Vishnubhog Mutant V-74-6	108.50	−10.70	107.64	−25.09	26.72	36.87	43.12	28.96
Vishnubhog Mutant V-47	111.00	−8.64	99.61	−30.68	18.20	−6.76	−2.52	−12.16
Vishnubhog Mutant V-45	103.67	−14.68	100.83	−29.83	22.73	16.46	21.75	9.70
Vishnubhog Mutant V-33	113.67	−6.45	103.86	−27.72	24.16	23.75	29.41	16.60
Vishnubhog Mutant V-45-2	112.50	−7.41	127.48	−11.28	20.09	2.92	7.61	−3.04
Vishnubhog Mutant V-67	115.50	−4.94	127.48	−11.28	12.42	−36.36	−33.48	−40.06
Vishnubhog Mutant V-71-4	101.67	−16.32	100.08	−30.35	26.34	34.93	41.08	27.12
Vishnubhog Mutant V-80	100.67	−17.15	113.02	−21.35	21.26	8.89	13.87	2.61
**Jhilli Dhan Parent**	**119.17**		**160.56**		**20.86**			
Jhilli Mutant J-2-13	93.67	−21.40	102.14	−36.38	19.54	−6.32	4.66	−5.69
Jhilli Mutant J-12-1	95.83	−19.58	101.71	−36.65	23.10	10.78	23.73	11.49
Jhilli Mutant J-13-2	98.67	−17.20	103.36	−35.62	19.98	−4.22	7.02	−3.57
Jhilli Dhan J-13-5	95.50	−19.86	106.79	−33.48	26.27	25.96	40.71	26.79
Jhilli Mutant J-15-1	95.50	−19.86	100.10	−37.65	24.09	15.51	29.03	16.26
**Dubraj Selection-1**	-	-	-	-	18.67	-	-	-
**Vishnubhog Selection-1**	-	-	-	-	20.72	-	-	-

**Table 3:** Analysis of variance (ANOVA) for agro-morphological and grain quality traits based on Factorial Randomized Complete Block Design.

Source of Variation	Replication	Factor A (Genotypes)	Factor B (Years)	Interaction A × B	Error	Total	CD for A	CD for B	CD for A × B	SE(m) for A × B
DF	1	23	2	46	71	143				
DFF	0.01	610.5 **	2124.3 **	43.6 **	6.53	2785	2.95	1.04	5.11	1.81
PH	4.91	2647.1 **	2262 **	62.84 **	4.49	4981	2.45	0.86	4.23	1.5
PL	0.5	50.23 **	22.36 **	5.07 **	0.91	144	0.55	0.2	0.95	0.68
FLL	0.75	102.16 **	28.90 **	7.02 **	2.11	140.9	1.68	0.59	2.9	1.03
FLW	0.01	0.52 **	0.10 **	0.02 **	0.01	0.66	0.11	0.04	0.18	0.07
TTP	3.14	306.41 **	67.45 **	6.15 **	1.57	145	1.45	0.51	2.5	0.89
ETP	47.32	93.30 **	123.91 **	11.61 **	5.01	281.5	2.58	0.91	4.47	1.58
FSP	126.9	5978.0 **	117.81 *	495.7 **	36.6	6755	6.98	2.47	12.09	4.28
SSP	7.51	213.48 **	127.42 **	111.2 **	21.5	146	5.35	1.89	9.27	3.28
TSP	196.44	5657.9 **	277.25 *	792.2 **	75.2	6999	10	3.54	17.32	6.13
SF%	0.62	173.41 **	16.93 *	15.34 **	3.51	209.8	2.16	0.76	3.74	1.32
HSW	0.01	1.58 **	0.33 **	0.04 **	0.01	147	0.13	0.05	0.23	0.08
GYP	1.19	111.49 **	6.40 *	3.06 *	1.68	123.8	1.5	0.53	2.59	0.92
Hul%	20.14	34.56 **	19.48 *	13.11 **	6.2	93.49	2.87	1.02	4.98	1.76
Mil%	6.78	27.02 **	57.23 **	13.95 **	5.46	148	2.7	0.95	4.67	1.65
HRR%	6.3	52.62 **	88.58 **	11.72	7.69	166.9	3.2	1.13	N/A	1.96
PadL	0.22	8.68 **	0.01	0.01	0.04	8.94	0.24	N/A	N/A	0.15
PadW	0.01	0.57 **	0.01	0.01	0.01	149	0.09	N/A	N/A	0.06
BRL	0.12	5.89 **	0.19 **	0.10 **	0.01	6.32	0.13	0.04	0.22	0.08
BRW	0.05	0.23 **	0.04 **	0.03 **	0.01	0.34	0.1	0.04	0.17	0.06
KL	0.02	5.09 **	1.73 **	0.20 **	0.01	150	0.12	0.04	0.21	0.07
KW	0.02	0.22 **	0.16 **	0.02 **	0.01	0.44	0.11	0.04	0.19	0.07
KLBR	0.01	1.96 **	0.05	0.07 **	0.02	2.11	0.16	N/A	0.28	0.1
CRL	0.08	7.86 **	8.30 **	0.61 **	0.03	151	0.19	0.07	0.33	0.12
CRW	0.06	0.37 **	0.04	0.06 *	0.03	0.55	0.2	N/A	0.35	0.12
ER	0.01	0.19 **	0.06 **	0.04 **	0	0.29	0.06	0.02	0.1	0.04
ASV	0.84	9.13 **	0.34 *	0.20 *	0.11	152	0.38	0.13	0.66	0.23
GCV	43.34	1043.83 **	1348.53 **	63.83 **	35.7	2535	6.9	2.44	11.94	4.23
AC	0.88	35.71 **	5.46 *	3.01 *	1.62	46.68	1.47	0.52	2.55	0.9

* Significant at 5% level of significance (*p* = 0.05); ** significant at 1% level of significance (*p* = 0.01).

**Table 4:** Parameters of the genetic variability based on the agro-morphological and grain quality traits.

Characters	Mean			Genotypic Coefficient of Variation (GCV)			Phenotypic Coefficient of Variation (GCV)			Heritability (%) (bs)			Genetic Advance as % Mean		
	*Kharif* 2019	*Rabi* 2019–20	*Kharif* 2020	*Kharif* 2019	*Rabi* 2019–20	*Kharif* 2020	*Kharif* 2019	*Rabi* 2019–20	*Kharif* 2020	*Kharif* 2019	*Rabi* 2019–20	*Kharif* 2020	*Kharif* 2019	*Rabi* 2019–20	*Kharif* 2020
Days to 50% flowering	105.25	117.1	106.1	11.25	5.89	11.65	11.56	6.29	11.83	94.74	87.69	97.06	22.56	20.37	23.64
Plant height (cm)	121.35	108.6	119.5	19.74	16.80	18.25	19.84	16.90	18.32	98.97	98.85	99.21	40.46	34.41	37.44
Panicle length (cm)	25.23	23.87	24.41	11.32	13.42	13.08	12.04	13.99	13.62	88.36	92.04	92.27	21.92	26.52	25.88
Flag leaf length (cm)	31.62	30.27	31.60	13.41	13.91	13.95	14.26	14.37	14.80	88.34	93.72	88.80	25.96	27.73	27.07
Flag leaf width (cm)	1.46	1.37	1.42	21.22	21.04	21.49	23.45	21.17	21.98	81.82	98.71	95.66	39.53	43.05	43.31
Total tillers/plant	12.08	13.96	11.75	53.81	55.19	65.88	54.59	56.08	66.77	97.17	96.84	97.35	109.2	111.8	133.91
Effective tillers/plant	10.83	13.04	10.00	42.38	23.34	48.15	44.23	31.96	48.91	91.83	53.34	96.93	83.66	35.11	97.67
Fertile spikelets/panicle	161.54	159.6	158.4	23.35	21.24	18.76	23.77	21.45	19.16	96.50	98.03	95.90	47.26	43.32	37.85
Sterile spikelets/panicle	47.38	44.54	47.46	24.06	8.20	13.95	25.98	14.25	16.48	85.77	33.14	71.64	45.90	9.73	24.32
Total spikelets/panicle	208.79	203.9	205.7	19.41	13.59	13.59	19.95	14.23	14.23	94.64	91.12	91.12	38.90	26.71	26.71
Spikelet Fertility (%)	76.83	77.65	76.50	8.48	7.44	7.44	8.86	7.71	7.71	91.74	93.23	93.23	16.74	14.80	14.80
100-Seed weight (g)	1.55	1.46	1.62	33.44	33.56	33.76	34.87	33.88	34.25	91.94	98.15	97.13	66.05	68.49	68.53
Grain yield/plant (g)	21.48	21.42	22.08	19.98	20.20	19.79	20.82	21.12	20.71	92.06	91.44	91.33	39.49	39.79	38.96
Hulling %	76.43	76.50	75.36	3.10	3.29	4.19	5.48	4.03	4.56	32.06	66.85	84.34	3.62	5.55	7.92
Milling %	67.57	67.16	65.50	3.86	3.83	3.96	5.72	4.63	4.92	45.60	68.53	64.75	5.37	6.53	6.57
Head Rice Recovery (%)	59.07	58.70	56.40	3.85	4.29	6.96	5.41	7.40	7.63	50.70	33.60	83.18	5.65	5.12	13.07
Paddy length (mm)	6.93	6.91	6.91	17.52	17.33	16.92	17.62	17.75	17.20	98.86	95.31	96.74	35.88	34.85	34.28
Paddy width (mm)	2.59	2.58	2.58	11.20	12.02	12.01	12.03	12.24	12.19	86.54	96.38	97.01	21.45	24.30	24.36
Dehusked rice length(mm)	5.21	5.18	5.09	19.86	19.09	19.50	19.93	19.22	19.62	99.31	98.70	98.84	40.77	39.07	39.94
Dehusked rice width (mm)	2.15	2.12	2.09	9.43	9.93	10.04	10.21	10.68	10.61	85.22	86.50	89.62	17.93	19.02	19.58
Milled rice length (mm)	4.90	4.88	4.56	20.90	19.47	20.88	21.02	19.57	22.57	98.80	98.96	85.65	42.78	39.90	39.81
Milled rice width (mm)	2.02	2.00	1.91	10.35	10.06	9.82	11.64	10.97	10.76	79.08	84.23	83.27	18.96	19.03	18.45
Kernel L/B ratio	2.46	2.48	2.42	23.29	24.29	24.31	24.20	24.75	24.96	92.63	96.35	94.88	46.17	49.12	48.78
Cooked rice length (mm)	7.46	7.45	6.74	20.91	14.96	15.09	21.10	15.10	15.22	98.19	98.21	98.40	42.68	30.54	30.84
Cooked rice width (mm)	2.88	2.86	2.91	9.68	9.50	9.51	11.74	10.33	10.34	68.02	84.60	84.65	16.45	17.99	18.03
Elongation ratio	1.63	1.55	1.49	19.50	19.79	18.70	20.61	20.11	19.38	89.60	96.85	93.14	38.03	40.13	37.18
Alkali spreading value	3.79	3.88	3.71	35.82	32.26	32.26	36.88	33.52	33.52	94.31	92.66	92.66	71.66	63.98	63.98
Gel consistency value (mm)	61.52	60.94	52.06	27.15	21.14	20.93	28.83	24.33	22.69	88.73	75.46	85.11	52.69	37.82	39.78
Amylose content (%)	24.54	24.00	23.92	9.18	10.44	11.04	11.02	11.56	12.01	69.28	81.43	84.43	15.73	19.40	19.89

**Table 5:** Path coefficient analysis for the agro-morphological traits during all three seasons.

Traits	Season	DFF	PH	PL	FLL	FLW	TTP	ETP	FSP	SSP	TSP	SF%	HSW	GYP
DFF	Kh19	**0.315**	0.074	0.005	−0.023	−0.034	0.013	0.008	−0.038	0.015	−0.038	−0.030	−0.066	−0.228 ^NS^
	Rb20	**0.56**	0.40	0.13	−0.03	−0.12	−0.03	−0.08	0.05	0.27	−0.15	−0.14	−0.28	−0.269 ^NS^
	Kh20	**0.39**	0.12	0.07	−0.01	−0.08	0.05	0.03	0.01	0.13	0.04	−0.09	−0.12	−0.151 ^NS^
PH	Kh19	−0.043	**−0.37**	−0.026	−0.008	0.016	0.005	0.006	0.011	0.013	0.015	−0.002	0.005	−0.019 *
	Rb20	−0.19	**−0.26**	−0.13	−0.08	0.00	0.05	0.10	−0.09	0.05	0.03	−0.05	0.00	−0.156 *
	Kh20	−0.23	**−0.36**	−0.15	−0.12	0.05	0.05	0.06	−0.06	−0.12	−0.09	0.02	0.01	−0.046 *
PL	Kh19	0.012	0.112	**0.294**	−0.047	−0.001	−0.039	−0.039	0.110	−0.138	0.086	0.148	0.021	0.696 **
	Rb20	0.08	0.17	**0.34**	−0.05	−0.08	0.00	−0.02	0.19	−0.10	0.07	0.14	−0.07	0.444 **
	Kh20	0.24	0.29	**0.69**	−0.19	−0.19	0.04	−0.01	0.30	0.11	0.31	0.14	−0.16	0.631 **
FLL	Kh19	0.045	−0.027	0.036	**−0.221**	−0.052	0.056	0.052	−0.029	−0.012	−0.034	−0.025	−0.092	0.118 ^NS^
	Rb20	0.01	−0.05	0.03	**−0.16**	−0.06	0.03	0.00	−0.02	0.01	−0.02	−0.03	−0.05	0.187 ^NS^
	Kh20	0.00	0.02	−0.02	**0.07**	0.02	−0.01	−0.02	0.00	0.00	0.00	0.00	0.02	0.193 ^NS^
FLW	Kh19	−0.058	−0.047	−0.001	0.046	**0.194**	−0.138	−0.134	0.106	−0.016	0.110	0.086	0.036	0.326 *
	Rb20	−0.18	−0.01	−0.19	0.34	**0.83**	−0.73	−0.56	0.32	0.13	0.50	0.43	0.32	0.497 **
	Kh20	−0.11	−0.04	−0.08	0.07	**0.28**	−0.26	−0.25	0.11	0.03	0.11	0.09	0.08	0.198 ^NS^
TTP	Kh19	0.181	−0.109	−0.209	−0.397	−1.116	**1.56**	1.561	−0.833	0.106	−0.866	−0.765	−0.347	0.297 *
	Rb20	−0.03	−0.13	−0.01	−0.12	−0.56	**0.64**	0.58	−0.29	−0.01	−0.33	−0.32	−0.20	0.421 **
	Kh20	−0.07	0.04	−0.02	0.06	0.27	**0.30**	−0.29	0.16	0.07	0.17	0.10	0.07	0.253 *
ETP	Kh19	−0.085	0.101	0.153	0.273	0.805	−1.163	**−1.165**	0.552	−0.123	0.562	0.537	0.232	0.257 ^NS^
	Rb20	−0.04	−0.11	−0.01	0.00	−0.18	0.25	**0.27**	−0.09	−0.11	−0.12	−0.16	−0.11	0.339 *
	Kh20	0.10	−0.09	−0.01	−0.15	−0.50	0.55	**0.55**	−0.29	−0.18	−0.32	−0.14	−0.11	0.272 ^NS^
FSP	Kh19	−1.350	−0.669	1.510	0.535	2.208	−2.145	−1.909	**4.024**	−1.597	3.932	3.635	0.483	0.788 **
	Rb20	0.01	0.04	0.06	0.02	0.04	−0.05	−0.04	**0.31**	−0.02	0.07	0.08	0.00	0.817 **
	Kh20	−1.21	−0.614	1.498	0.518	2.540	−2.321	−1.531	**3.94**	−1.891	3.448	3.252	0.342	0.767 **
SSP	Kh19	0.172	−0.249	−0.617	0.073	−0.109	0.089	0.138	−0.519	**1.309**	−0.251	−0.929	−0.588	−0.770 **
	Rb20	−0.06	0.02	0.03	0.01	−0.02	0.001	0.05	0.02	**−0.12**	0.00	0.06	0.07	−0.473 **
	Kh20	0.158	−0.215	−0.512	0.061	−0.114	0.067	0.125	−0.473	**1.235**	−0.218	−0.889	−0.654	−0.235 *
TSP	Kh19	1.293	0.875	−1.151	−0.611	−2.236	2.184	1.903	−3.852	0.757	**3.942**	−3.157	−0.095	0.663 **
	Rb20	−0.08	−0.03	0.05	0.04	0.17	−0.15	−0.13	0.18	0.00	**0.28**	0.22	−0.01	0.791 **
	Kh20	1.124	0.745	−1.17	−0.521	−2.324	2.047	1.835	−3.355	0.698	**3.652**	−3.864	−0.087	0.644 **
SF%	Kh19	−0.357	0.044	0.685	0.154	0.604	−0.665	−0.627	1.227	−0.964	1.088	**1.359**	0.411	0.934 **
	Rb20	−0.14	0.12	0.24	0.11	0.31	−0.30	−0.35	0.45	−0.29	0.48	**0.60**	0.19	0.971 **
	Kh20	−0.21	−0.02	0.09	0.02	0.16	−0.15	−0.12	0.32	−0.29	0.21	**0.47**	0.19	0.761 **
HSW	Kh19	−0.154	−0.019	0.019	0.111	0.049	−0.060	−0.054	0.032	−0.121	0.006	0.081	**0.269**	0.268 *
	Rb20	−0.22	−0.01	−0.10	0.13	0.17	−0.14	−0.17	−0.01	−0.29	−0.01	0.14	**0.44**	0.308 *
	Kh20	−0.18	−0.01	−0.07	0.08	0.08	−0.07	−0.06	−0.01	−0.17	−0.06	0.12	**0.29**	0.166 *
GYP	Kh19	−0.228 ^NS^	−0.019 *	0.696 **	0.118 ^NS^	0.326 *	0.297 *	0.257 ^NS^	0.788 **	−0.77 **	0.663 **	0.934 **	0.268 *	-
	Rb20	−0.26 ^NS^	−0.156 *	0.444 **	0.187 ^NS^	0.497 **	0.42 **	0.339 *	0.817 **	−0.47 **	0.791 **	0.971 **	0.308 *	-
	Kh20	−0.15 ^NS^	−0.046 *	0.631 **	0.19 ^NS^	0.198 ^NS^	0.25 *	0.27 ^NS^	0.767 **	−0.23 *	0.644 **	0.761 **	0.166 *	-

Bold values (figures) present in diagonal blocks of the table are the direct effects of each trait for the dependent variable (HRR%). NB: Kh19 = *Kharif* 2019; Rb20 = *Rabi* 2019–20; Kh20 = *Kharif* 2020. * Significant at 5% level of significance (*p* = 0.05); ** significant at 1% level of significance (*p* = 0.01); NS: no significance.

**Table 6:** Path coefficient analysis for the grain quality traits during all three seasons.

Traits	Seasons	Hul (%)	Mil (%)	PadL	PadB	BRL	BRB	KL	KB	KLBR	CRL	CRW	ER	GC	AC (%)	HRR (%)
Hul (%)	Kh19	**0.50**	0.41	0.17	−0.24	0.16	−0.11	0.06	−0.09	0.12	0.06	−0.18	0.15	−0.17	0.08	0.353 *
	Rb20	**1.53**	−1.28	−0.74	0.81	−0.57	0.65	−0.42	0.57	−0.58	−0.21	0.76	−0.56	0.55	−0.14	0.778 **
	Kh20	**0.66**	0.44	−0.07	0.01	−0.07	0.04	−0.09	0.18	−0.15	−0.19	0.01	−0.21	−0.24	0.35	0.871 **
Mil (%)	Kh19	0.10	**0.12**	0.06	−0.04	0.05	−0.02	0.03	−0.02	0.04	0.02	−0.05	0.05	−0.04	0.04	0.593 **
	Rb20	0.96	**1.16**	0.63	−0.42	0.40	−0.29	0.34	−0.25	0.40	0.23	−0.56	0.47	−0.39	0.19	0.908 **
	Kh20	0.20	**0.30**	0.01	0.04	0.02	0.01	−0.03	0.03	−0.04	−0.03	0.00	−0.03	−0.12	0.10	0.644 **
PadL	Kh19	0.18	0.28	**0.52**	−0.12	0.49	−0.14	0.46	−0.13	0.47	0.37	−0.29	0.45	−0.14	0.10	−0.105 ^NS^
	Rb20	−0.79	−0.90	**−1.65**	0.39	−1.55	0.38	−1.53	0.43	−1.44	−1.16	0.90	−1.41	0.47	−0.28	−0.168 ^NS^
	Kh20	−0.11	0.02	**1.05**	−0.28	0.98	−0.03	0.82	−0.19	0.81	0.78	0.13	0.81	−0.12	−0.01	−0.261 ^NS^
PadB	Kh19	−0.05	−0.03	−0.02	**0.10**	−0.03	0.07	−0.03	0.07	−0.04	0.01	0.06	−0.02	0.04	−0.02	−0.220 ^NS^
	Rb20	−0.21	−0.14	−0.09	**0.40**	−0.11	0.27	−0.09	0.26	−0.19	0.05	0.23	−0.08	0.12	−0.04	−0.440 **
	Kh20	−0.01	−0.10	0.21	**−0.81**	0.26	−0.58	0.21	−0.70	0.50	−0.06	−0.46	0.13	−0.21	0.07	−0.119 ^NS^
BRL	Kh19	−0.85	−1.02	−2.40	0.75	**−2.55**	0.69	−2.47	0.63	−2.38	−1.88	1.26	−2.18	0.54	−0.54	−0.236 ^NS^
	Rb20	2.24	2.04	5.59	−1.72	**5.97**	−1.43	5.83	−1.79	5.47	4.29	−2.85	4.83	−1.37	0.98	−0.378 **
	Kh20	0.26	−0.20	−2.29	0.78	**−2.45**	0.02	−2.15	0.35	−2.08	−1.96	−0.44	−1.96	0.57	−0.16	−0.246 ^NS^
BRB	Kh19	−0.10	−0.06	−0.12	0.29	−0.12	**0.45**	−0.11	0.45	−0.24	0.03	0.29	−0.12	0.03	0.11	−0.098 ^NS^
	Rb20	2.49	1.48	1.35	−3.99	1.41	**−5.88**	1.03	−5.73	3.26	−0.66	−3.96	1.70	−0.51	−1.22	−0.357 *
	Kh20	−0.06	−0.02	0.03	−0.62	0.01	**−0.87**	−0.08	−0.86	0.28	−0.25	−0.35	−0.14	0.15	−0.28	−0.078 ^NS^
KL	Kh19	0.14	0.31	1.06	−0.32	1.16	−0.30	**1.20**	−0.29	1.13	0.88	−0.55	0.99	−0.27	0.24	−0.285 *
	Rb20	−0.92	−0.99	−3.12	0.79	−3.30	0.59	**−3.38**	0.79	−3.06	−2.37	1.61	−2.68	0.51	−0.70	−0.428 **
	Kh20	−0.28	−0.17	1.51	−0.51	1.71	0.19	**1.95**	−0.05	1.77	1.51	0.44	1.47	−0.51	0.35	−0.292 *
KB	Kh19	0.02	0.02	0.03	−0.08	0.03	−0.12	0.03	**−0.12**	0.07	−0.01	−0.09	0.03	−0.02	−0.02	−0.139 ^NS^
	Rb20	−2.35	−1.38	−1.63	4.11	−1.89	6.13	−1.47	**6.29**	−3.85	0.48	4.69	−2.20	0.65	1.14	−0.273 ^NS^
	Kh20	0.13	0.05	−0.09	0.42	−0.07	0.48	−0.01	**0.49**	−0.21	0.08	0.20	0.00	−0.06	0.14	0.016 ^NS^
KLBR	Kh19	0.12	0.19	0.45	−0.22	0.47	−0.27	0.47	−0.27	**0.50**	0.29	−0.35	0.40	−0.08	0.07	−0.140 ^NS^
	Rb20	−0.94	−0.85	−2.14	1.15	−2.25	1.36	−2.23	1.50	**−2.46**	−1.28	1.75	−1.95	0.39	−0.20	−0.224 ^NS^
	Kh20	0.45	0.24	−1.54	1.24	−1.69	0.63	−1.81	0.85	**−1.99**	−1.20	−0.01	−1.31	0.36	−0.08	−0.246 ^NS^
CRL	Kh19	−0.11	−0.15	−0.69	−0.12	−0.72	−0.07	−0.71	−0.11	−0.56	**−0.98**	0.11	−0.83	0.17	−0.17	−0.471 **
	Rb20	0.40	0.56	2.00	0.35	2.05	0.32	2.00	0.22	1.49	**2.85**	−0.31	2.39	−0.59	0.61	−0.549 **
	Kh20	−0.67	−0.24	1.70	0.17	1.83	0.66	1.77	0.38	1.38	**2.29**	1.00	2.07	0.06	−0.06	−0.503 **
CRW	Kh19	−0.01	−0.01	−0.02	0.02	−0.02	0.02	−0.01	0.02	−0.02	0.00	**−0.03**	−0.02	0.01	0.00	−0.321 *
	Rb20	2.54	2.49	2.78	−2.94	2.44	−3.44	2.43	−3.80	3.64	0.56	**−5.11**	3.19	−1.16	0.69	−0.404 **
	Kh20	−0.01	0.00	−0.08	−0.38	−0.12	−0.27	−0.15	−0.28	0.00	−0.29	**−0.67**	0.01	−0.10	0.05	−0.148 ^NS^
ER	Kh19	0.25	0.34	0.69	−0.16	0.69	−0.22	0.66	−0.22	0.65	0.68	−0.50	**0.81**	−0.15	0.15	−0.200 ^NS^
	Rb20	−1.48	−1.63	−3.42	0.78	−3.26	1.16	−3.20	1.41	−3.20	−3.37	2.52	**−4.02**	1.01	−0.89	−0.227 ^NS^
	Kh20	0.49	0.13	−1.20	0.24	−1.25	−0.25	−1.17	−0.01	−1.03	−1.40	0.01	**−1.56**	0.10	−0.06	−0.493 **
GC	Kh19	0.01	0.01	0.01	−0.01	0.01	0.00	0.01	−0.01	0.01	0.01	−0.01	0.01	**−0.04**	0.03	−0.163 ^NS^
	Rb20	−0.09	−0.08	−0.07	0.07	−0.05	0.02	−0.04	0.02	−0.04	−0.05	0.05	−0.06	**0.24**	−0.17	−0.136 ^NS^
	Kh20	0.60	0.67	0.19	−0.44	0.39	0.28	0.44	0.22	0.30	−0.05	−0.25	0.10	**−1.68**	1.45	−0.599 **
AC (%)	Kh19	−0.01	−0.03	−0.01	0.01	−0.01	−0.02	−0.01	−0.01	−0.01	−0.01	0.01	−0.01	0.04	**−0.07**	0.038 ^NS^
	Rb20	−0.03	−0.05	−0.05	0.03	−0.05	−0.06	−0.06	−0.05	−0.02	−0.06	0.04	−0.06	0.20	**−0.29**	−0.273 ^NS^
	Kh20	−0.78	−0.47	0.01	0.12	−0.10	−0.47	−0.26	−0.44	−0.06	0.04	0.12	−0.06	1.26	**−1.47**	0.453 **
HRR (%)	Kh19	0.35 *	0.59 **	−0.11 ^NS^	−0.22 ^NS^	−0.24 ^NS^	−0.098 ^NS^	−0.285 *	−0.14 ^NS^	−0.140 ^NS^	−0.471 **	−0.321 *	−0.200 ^NS^	−0.163 ^NS^	0.038 ^NS^	-
	Rb20	0.778 **	0.908 **	−0.168 ^NS^	−0.440 **	−0.378 **	−0.357 *	−0.428 **	−0.273 ^NS^	−0.224 ^NS^	−0.549 **	−0.404 **	−0.227 ^NS^	−0.136 ^NS^	−0.273 ^NS^	-
	Kh20	0.871 **	0.644 **	−0.261 ^NS^	−0.119 ^NS^	−0.246 ^NS^	−0.078 ^NS^	−0.292 *	0.016 ^NS^	−0.246 ^NS^	−0.503 **	−0.14 ^NS^	−0.493 **	−0.599 **	0.453 **	-

Bold values (figures) present in diagonal blocks of the table are the direct effects of each traits for the dependent variable (HRR%). NB: Kh19 = *Kharif* 2019; Rb20 = *Rabi* 2019–20; Kh20 = *Kharif* 2020. * Significant at 5% level of significance (*p* = 0.05); ** significant at 1% level of significance (*p* = 0.01); NS: no significance.

**Table 7:** Informativeness and profiles of the 46 SSR markers taken for the present study.

Marker	Chromosome No.	Position (cM)	Forward Primer	Reverse Primer	Major Allele Frequency	Genotype No.	Allele No.	Gene Diversity	PIC
RM 283	1	31.4	gtctacatgtacccttgttggg	cggcatgagagtctgtgatg	0.417	3.000	3.000	0.642	0.566
RM 259	1	54.2	tggagtttgagaggaggg	cttgttgcatggtgccatgt	0.750	3.000	3.000	0.403	0.363
RM 312	1	71.6	gtatgcatatttgataagag	aagtcaccgagtttaccttc	0.417	3.000	3.000	0.642	0.566
RM 5	1	94.9	tgcaacttctagctgctcga	gcatccgatcttgatggg	0.500	3.000	3.000	0.625	0.555
RM 237	1	115.2	caaatcccgactgctgtcc	tgggaagagagcactacagc	0.708	2.000	2.000	0.413	0.328
RM 154	2	4.8	accctctccgcctcgcctcctc	ctcctcctcctgcgaccgctcc	0.375	3.000	3.000	0.663	0.589
RM 452	2	58.4	ctgatcgagagcgttaaggg	gggatcaaaccacgtttctg	1.000	1.000	1.000	0.000	0.000
RM 489	3	29.2	acttgagacgatcggacacc	tcacccatggatgttgtcag	0.375	3.000	3.000	0.656	0.582
OSR-13	3	53.1	catttgtgcgtcacggagta	agccacagcgcccatctctc	0.476	5.000	5.000	0.667	0.614
RM 338	3	108.4	cacaggagcaggagaagagc	ggcaaaccgatcactcagtc	0.500	2.000	2.000	0.500	0.375
RM 55	3	168.2	ccgtcgccgtagtagagaag	tcccggttattttaaggcg	0.381	5.000	5.000	0.726	0.679
RM 514	3	216.4	agattgatctcccattcccc	cacgagcatattactagtgg	0.500	3.000	3.000	0.625	0.555
RM 307	4	0	gtactaccgacctaccgttcac	ctgctatgcatgaactgctc	0.600	2.000	2.000	0.480	0.365
RM 124	4	150.1	atcgtctgcgttgcggctgctg	catggatcaccgagctcccccc	0.917	2.000	2.000	0.153	0.141
RM 507	5	0	cttaagctccagccgaaatg	ctcaccctcatcatcgcc	0.913	2.000	2.000	0.159	0.146
RM 161	5	96.9	tgcagatgagaagcggcgcctc	tgtgtcatcagacggcgctccg	0.739	2.000	2.000	0.386	0.311
RM 178	5	118.8	tcgcgtgaaagataagcggcgc	gatcaccgttccctccgcctgc	0.792	2.000	2.000	0.330	0.275
RM 133	6	0	ttggattgttttgctggctcgc	ggaacacggggtcggaagcgac	0.458	3.000	3.000	0.601	0.516
RM 510	6	20.8	aaccggattagtttctcgcc	tgaggacgacgagcagattc	0.833	2.000	2.000	0.278	0.239
RM 454	6	99.3	ctcaagcttagctgctgctg	gtgatcagtgcaccatagcg	0.958	2.000	2.000	0.080	0.077
RM 162	6	108.3	gccagcaaaaccagggatccgg	caaggtcttgtgcggcttgcgg	1.000	1.000	1.000	0.000	0.000
RM 455	7	65.7	aacaacccaccacctgtctc	agaaggaaaagggctcgatc	0.750	2.000	2.000	0.375	0.305
RM 118	7	96.9	ccaatcggagccaccggagagc	cacatcctccagcgacgccgag	0.750	2.000	2.000	0.375	0.305
RM 408	8	0	caacgagctaacttccgtcc	actgctacttgggtagctgacc	0.375	4.000	4.000	0.729	0.681
RM 152	8	9.4	gaaaccaccacacctcaccg	ccgtagaccttcttgaagtag	0.750	2.000	2.000	0.375	0.305
RM 44	8	60.9	acgggcaatccgaacaacc	tcgggaaaacctaccctacc	0.550	2.000	2.000	0.495	0.372
RM 284	8	83.7	atctctgatactccatccatcc	cctgtacgttgatccgaagc	0.667	2.000	2.000	0.444	0.346
RM 433	8	116	tgcgctgaactaaacacagc	agacaaacctggccattcac	0.905	2.000	2.000	0.172	0.157
RM 447	8	124.6	cccttgtgctgtctcctctc	acgggcttcttctccttctc	0.542	3.000	3.000	0.531	0.428
RM 22579	8	5,972,576	tccactttacatcgtcacaa	ctacctcttaaccgcacatt	0.500	4.000	4.000	0.642	0.583
RM 3155	8	119.9	gtaactgtttcgcttgcttt	atctcatacccaatttcgtg	0.429	3.000	3.000	0.612	0.530
RM 316	9	1.8	ctagttgggcatacgatggc	acgcttatatgttacgtcaac	0.773	3.000	3.000	0.376	0.344
RM 105	9	32.1	gtcgtcgacccatcggagccac	tggtcgaggtggggatcgggtc	0.771	4.000	3.000	0.379	0.348
RM 215	9	99.4	caaaatggagcagcaagagc	tgagcacctccttctctgtag	0.714	2.000	2.000	0.408	0.325
RM 474	10	0	aagatgtacgggtggcattc	tatgagctggtgagcaatgg	0.458	3.000	3.000	0.642	0.570
RM 271	10	59.4	tcagatctacaattccatcc	tcggtgagacctagagagcc	0.417	4.000	4.000	0.670	0.606
RM 171	10	73	aacgcgaggacacgtacttac	acgagatacgtacgcctttg	0.900	2.000	2.000	0.180	0.164
RM 484	10	97.3	tctccctcctcaccattgtc	tgctgccctctctctctctc	1.000	1.000	1.000	0.000	0.000
RM 552	11	40.6	cgcagttgtggatttcagtg	tgctcaacgtttgactgtcc	1.000	1.000	1.000	0.000	0.000
RM 536	11	55.1	tctctcctcttgtttggctc	acacaccaacacgaccacac	0.833	2.000	2.000	0.278	0.239
RM 287	11	68.6	ttccctgttaagagagaaatc	gtgtatttggtgaaagcaac	0.500	4.000	4.000	0.656	0.605
RM 144	11	123.2	tgccctggcgcaaatttgatcc	gctagaggagatcagatggtagtgcatg	1.000	1.000	1.000	0.000	0.000
RM 19	12	20.9	caaaaacagagcagatgac	ctcaagatggacgccaaga	0.375	3.000	3.000	0.663	0.589
RM 277	12	57.2	cggtcaaatcatcacctgac	caaggcttgcaagggaag	0.750	2.000	2.000	0.375	0.305
RM 28107	12	1,6052,560	gaaatatttagttccggacg	taatcaaacctggaagagga	0.500	3.000	3.000	0.594	0.511
RM 2734	12	2,6153,565	tgttctggaggtaggtatgg	cagcaactcaaagtatgcaa	0.542	4.000	4.000	0.604	0.541
**Mean**					**0.660**	**2.587**	**2.565**	**0.426**	**0.370**

**Table 8:** Genomic similarities between the mutants and respective parents based on diverse SSR markers.

Marker	Chromosome No.	VB Parent	VBM 71-4	VBM-74-6	VBM 19-2	VBM 45-2	VBM 45	VBM 80	VBM 47	VBM 33	VBM 17	VBM 67	SC Parent	SCM 49	SCM 50	SCM 18-1	Jhilli Parent	Jhilli M13-5	Jhilli M2-13	Jhilli M13-2	Jhilli M15-1	Jhilli M 12-1
RM 283	1	A	A	B	B	B	A	A	A	B	A	A	A	A	A	A	A	B	A	A	A	A
RM 259	1	A	A	A	A	A	A	A	A	A	A	B	A	A	A	A	A	A	A	A	A	A
RM 312	1	A	A	A	A	A	A	A	A	A	A	A	A	A	A	B	A	A	A	A	A	A
RM 5	1	A	A	A	A	A	A	B	B	A	C	C	A	A	A	A	A	A	A	B	A	B
RM 237	1	A	A	B	A	A	A	A	B	B	A	A	A	A	A	A	A	A	A	A	A	A
RM 154	2	A	A	A	A	A	A	B	B	B	C	C	A	A	A	A	A	A	A	A	A	A
RM 452	2	A	A	A	A	A	A	A	A	A	A	A	A	A	A	A	A	A	A	A	A	A
RM 489	3	A	A	A	A	A	A	A	A	A	B	B	A	A	A	A	A	A	A	A	A	A
OSR-13	3	A	A	A	B	A	A	A	A	C	A	A	A	A	A	A	A	A	A	A	A	A
RM 338	3	A	NA	NA	NA	NA	NA	A	B	A	A	A	A	A	A	A	A	A	A	A	A	A
RM 55	3	A	A	A	A	A	NA	A	NA	NA	B	B	A	A	A	A	A	B	C	A	A	A
RM 514	3	A	A	A	A	A	A	A	A	A	A	A	A	A	A	A	A	B	A	B	A	B
RM 307	4	A	A	A	A	A	NA	A	A	A	A	B	A	NA	NA	A	A	A	A	A	A	A
RM 124	4	A	A	A	A	A	A	A	A	A	A	A	A	B	B	B	A	A	A	A	A	A
RM 507	5	A	A	A	A	B	A	A	A	A	A	A	A	A	A	A	A	A	A	A	A	A
RM 161	5	A	A	NA	A	A	A	A	A	A	A	A	A	A	A	A	A	A	A	A	A	A
RM 178	5	A	A	A	A	A	A	A	A	A	A	A	A	A	A	A	A	A	A	A	A	A
RM 133	6	A	A	A	A	A	A	A	A	A	A	A	A	A	A	A	A	A	A	A	A	A
RM 510	6	A	A	A	A	A	B	A	A	B	B	B	A	A	A	A	A	A	A	A	A	A
RM 454	6	A	A	A	B	A	A	A	A	A	A	A	A	A	A	A	A	A	A	A	A	A
RM 162	6	A	A	NA	NA	NA	NA	A	A	A	A	A	A	A	A	A	A	A	A	A	A	A
RM 455	7	A	A	A	A	A	A	A	A	A	A	A	A	A	A	A	A	A	A	B	B	B
RM 118	7	A	A	A	A	A	A	A	A	A	A	B	A	A	A	A	A	A	A	A	A	A
RM 408	8	A	A	A	A	A	A	A	B	B	A	A	A	A	A	A	A	B	B	B	C	C
RM 152	8	A	A	A	B	A	B	A	A	B	A	A	A	A	A	A	A	A	A	A	A	A
RM 44	8	A	B	NA	NA	NA	A	A	B	NA	B	B	A	A	A	A	A	A	A	A	A	A
RM 284	8	A	A	A	A	A	A	A	A	A	B	B	A	A	A	A	A	A	A	B	B	B
RM 433	8	A	NA	NA	NA	A	A	A	A	A	A	A	A	A	A	A	A	A	A	A	A	A
RM 447	8	A	A	A	A	B	A	A	A	B	A	A	A	A	A	A	A	A	A	A	A	A
RM 316	9	A	A	A	A	A	A	A	A	A	A	A	A	NA	B	B	A	A	A	B	A	A
RM 105	9	A	A	A	A	A	A	A	A	A	A	A	A	B	C	A	A	A	A	A	A	A
RM 215	9	A	A	A	A	A	A	B	A	A	A	A	A	NA	NA	B	A	A	A	A	A	B
RM 474	10	A	A	A	A	A	B	A	B	B	B	A	A	A	B	B	A	A	A	A	A	A
RM 271	10	A	A	A	A	A	A	A	A	A	A	A	A	A	A	A	A	A	A	A	A	A
RM 171	10	A	A	A	A	NA	NA	A	A	A	A	A	A	A	A	A	A	NA	NA	A	B	B
RM 484	10	A	A	A	A	A	A	A	A	A	A	A	A	A	A	A	A	A	A	A	A	A
RM 552	11	A	A	A	A	A	A	A	A	A	A	A	A	A	A	A	A	A	A	A	A	A
RM 536	11	A	A	A	A	A	A	A	A	A	B	A	A	A	A	A	A	A	A	A	A	A
RM 287	11	A	B	A	A	A	A	B	A	A	A	A	A	A	A	A	A	A	A	A	A	A
RM 144	11	A	A	A	A	A	A	A	A	A	A	A	A	A	A	A	A	A	A	A	A	A
RM 19	12	A	A	A	A	A	A	A	A	A	C	C	A	A	A	A	A	A	A	A	A	C
RM 277	12	A	A	A	A	A	A	A	A	A	B	B	A	A	A	A	A	A	A	A	A	A
Hv 8-14	8	A	A	A	A	A	A	A	A	A	A	A	A	A	A	A	A	A	A	A	A	A
Hv 8-50	8	A	A	A	A	A	A	A	A	A	C	C	A	NA	NA	A	A	A	A	A	B	B
Hv 12-28	12	A	A	A	A	A	A	A	A	A	A	A	A	A	A	A	A	A	A	A	A	A
Hv 12-46	12	A	A	A	A	A	A	A	A	A	A	A	A	A	A	A	A	A	A	A	A	A
Number of dissimilar alleles in mutants over the parent			**2**	**2**	**3**	**3**	**3**	**4**	**7**	**9**	**12**	**13**		**2**	**4**	**5**		**4**	**2**	**6**	**5**	**9**
Number of similar alleles in mutants over the parent			**44**	**44**	**43**	**43**	**43**	**42**	**39**	**37**	**34**	**33**		**44**	**42**	**41**		**42**	**44**	**39**	**41**	**37**
Genomic similarity (%) of mutants with parents			**95.6**	**95.6**	**93.4**	**93.4**	**93.4**	**91.3**	**84.7**	**80.4**	**73.9**	**71.7**		**95.6**	**91.3**	**89.1**		**91.3**	**95.6**	**84.7**	**89.1**	**80.4**

NB: (1) Coloured cells showed the dissimilar alleles of mutants as compared to their corresponding parents.

**Table 9:** List of experimental materials used in the current study.

S. No.	Name of the Experimental Materials	Codings	Parentage	Role
1	Vishnubhog *	VB parent	Local landrace	Parent
2	Samundchini *	SC Parent	Local landrace	Parent
3	Jhilli *	Jhilli Parent	Local landrace	Parent
4	Vishnubhog Mutant V-17 ^#^	VBM 17	Vishnubhog	Mutant
5	Vishnubhog Mutant V-19-2 ^#^	VBM 19-2	Vishnubhog	Mutant
6	Vishnubhog Mutant V-74-6 ^#^	VBM-74-6	Vishnubhog	Mutant
7	Vishnubhog Mutant V-47 ^#^	VBM 47	Vishnubhog	Mutant
8	Vishnubhog Mutant V-45 ^#^	VBM 45	Vishnubhog	Mutant
9	Vishnubhog Mutant V-33 ^#^	VBM 33	Vishnubhog	Mutant
10	Vishnubhog Mutant V-45-2 ^#^	VBM 45-2	Vishnubhog	Mutant
11	Vishnubhog Mutant V-67 ^#^	VBM 67	Vishnubhog	Mutant
12	Vishnubhog Mutant V-71-4 ^#^	VBM 71-4	Vishnubhog	Mutant
13	Vishnubhog Mutant V-80 ^#^	VBM 80	Vishnubhog	Mutant
14	Samundchini Mutant S-49 ^#^	SCM 49	Samundchini	Mutant
15	Samundchini Mutant S-18-1 ^#^	SCM 18-1	Samundchini	Mutant
16	Samundchini Mutant S-50 ^#^	SCM 50	Samundchini	Mutant
17	Jhilli Mutant J-2-13 ^#^	Jhilli M2-13	Jhilli	Mutant
18	Jhilli Mutant J-12-1 ^#^	Jhilli M12-1	Jhilli	Mutant
19	Jhilli Mutant J-13-2 ^#^	Jhilli M13-2	Jhilli	Mutant
20	Jhilli Dhan J-13-5 ^#^	Jhilli M13-5	Jhilli	Mutant
21	Jhilli Mutant J-15-1 ^#^	Jhilli M15-1	Jhilli	Mutant
22	Dubraj selection -1 *	Dub Sel.-1	Dubraj	Check
23	Vishnubhog Selection-1 *	VB Sel.-1	Vishnubhog	Check
24	Rajeshwari *	Rajes.	R320-300 x Chepti Gurmatiya	Check

* These genotypes were procured from the R.H. Richharia Rice Germplasm Division of the Department of Genetics and Plant Breeding, Indira Gandhi Krishi Vishwavidyalaya, Raipur (C.G.), India; ^#^ Mutant lines generated from their respective parental landraces.

## Data Availability

Not applicable.

## Supplementary Materials

The following supporting information can be downloaded at: https://www.mdpi.com/article/10.3390/plants11243448/s1: Figure S1: Field layout plan of Randomized Complete Block Design for 24 genotypes, including 18 mutants, 3 parents and 3 checks, during all three seasons. Table S1: List of putative mutants identified and selected in the M2 population of Jhilli, Samundchini and Vishnubhog. Table S2: Mean performance of 24 rice genotypes for 13 agro-morphological traits taken during three consecutive seasons/generations. Table S3: Mean performance of 24 rice genotypes for 18 grain quality traits taken during three consecutive seasons/generations. Table S4: Shapiro–Wilk W test of the agro-morphological and grain quality traits during three consecutive seasons. Table S5. Correlation coefficients for agro-morphological traits during all three seasons. Table S6: Correlation coefficients for grain quality traits during all three seasons. Table S7: Details of the three rice landraces used in the current study.
