# Supplementary material for: Genetic Improvement in Plant Architecture, Maturity Duration and Agronomic Traits of Three Traditional Rice Landraces through Gamma Ray-Based Induced Mutagenesis
Source: Plants (Basel). 2022 Dec 9;11(24):3448. doi: 10.3390/plants11243448 (PMC9781505; doi:10.3390/plants11243448)
Supplement: Supplementary file 1 [file plants-11-03448-s001.zip › plants-2003286-supplementary.pdf]

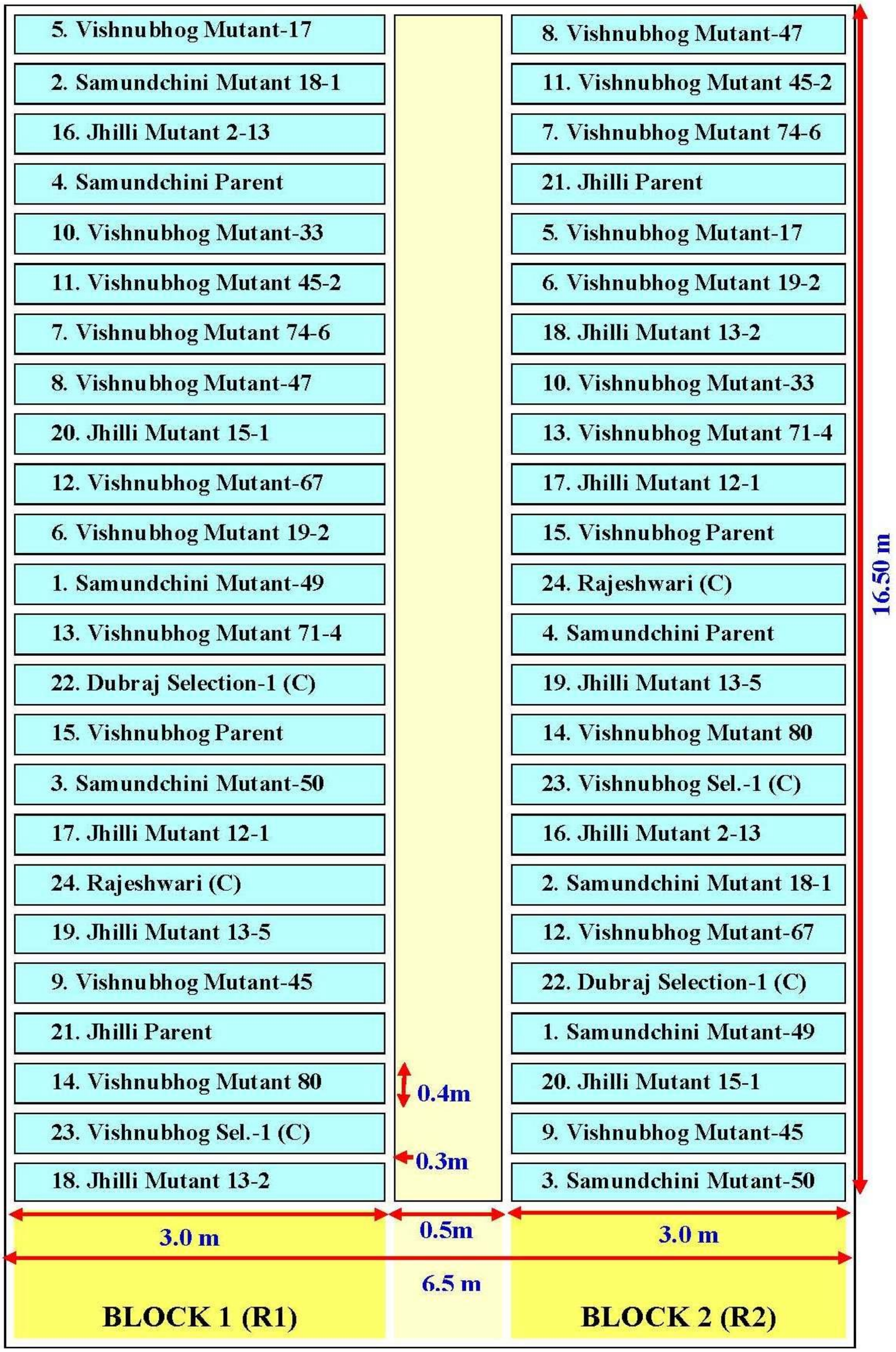

Supplementary Figure S1: Field Layout Plan of Randomized Complete Block Design for 24 Genotypes including 18 Mutants, 3 Parents and 3 Checks during all three seasons

Supplementary Table S1: List of putative mutants identified and selected in M_2 population of Jhilli, Samundchini and Vishnubhog

Type of macro mutants	**Jhilli**		**Samundchini**		**Vishnubhog**	
	Categories of putative mutants	Number of mutants in M_2 population	Categories of putative mutants	Number of mutants in M_2 population	Categories of putative mutants	Number of mutants in M_2 population
Chlorophyll mutants	Albino	17	Albino	28	Albino	23
	Xantha	9	Xantha	16	Xantha	13
	Chlorina	5	Chlorina	9	Chlorina	7
	Virdis	1	Virdis	3	Virdis	2
	**Total chlorophyll mutants**	**32**	**Total chlorophyll mutants**	**56**	**Total chlorophyll mutants**	**45**
Morphological mutants	Grassy mutant	4	Grassy mutants	9	Grassy mutants	5
	Broad leaf mutants	1	Broad and dark green leaf mutants	2	High tillering mutants	5
	High tillering and vigorous plant	1	Narrow and dark green leaf mutants	4	Broad leaf, high tillering, vigorous mutant	2
	Dwarf and mid early	5	High tillering and vigorous mutants	2	Narrow leaf mutant	1
	Semi-dwarf and early	4	Erect leaves and sturdy stem mutants	1	Purple leaf mutant	3
	Semi dwarf and mid early	2	Strong stem and semi dwarf mutants	1	Dark green leaf mutant	1
	Clustered grain	2	Dwarf and mid early	7	Mid early to early mutants	5
	**Total morphological mutants**	**19**	Semi dwarf and mid early	8	Dwarf mutants	4
			Semitall and mid early mutants	2	Semi dwarf mid early mutants	4
			Semi dwarf and late mutants	2	Semi dwarf and late mutants	3
			Fine grain mutants	2	Semitall mutants	3
			Clustered grain mutant	2	**Total morphological mutants**	**36**
			**Total morphological mutants**	**42**		
	**Total putative macro mutants in Jhilli**	**51**	**Total putative macro mutants in Samundchini**	**98**	**Total putative macro mutants in Vishnubhog**	**81**

Supplementary Table S2: Mean performance of 24 rice genotypes for 13 agro-morphological traits taken during three consecutive seasons/ generations

Genotypes	Season/Year	Generation	DFF	PH	PL	FLL	FLW	TTP	ETP	FSP	SSP	TSP	SF%	HSW	GYP
Samundchini Mutant S-49	*Kharif* 2019	M_4	117	117.48	25.80	25.25	0.63	38	28	133	50	182	72.81	0.88	22.11
	Rabi 2019-20	M_5	118	107.25	26.68	23.83	0.48	39	17	111	45	156	70.94	0.86	18.15
	Kharif 2020	M_6	121	117.05	27.58	25.93	0.45	34	25	109	42	151	72.41	0.85	21.50
Samundchini Mutant S-18-1	*Kharif* 2019	M_4	114	121.50	30.25	24.98	1.45	10	9	184	39	223	82.77	1.01	27.23
	Rabi 2019-20	M_5	119	113.75	30.58	22.25	1.33	11	13	226	43	269	84.19	1.03	27.11
	Kharif 2020	M_6	118	121.90	28.58	25.14	1.22	10	9	243	59	302	80.63	0.97	30.49
Samundchini Mutant S-50	*Kharif* 2019	M_4	116	124.50	29.35	24.05	1.43	9	8	171	45	216	79.19	1.00	27.15
	Rabi 2019-20	M_5	119	118.00	30.30	26.45	1.15	10	13	195	40	234	83.12	1.04	23.02
	Kharif 2020	M_6	118	124.25	29.08	25.75	1.26	10	9	198	55	253	78.37	0.96	27.64
Samundchini Parent	*Kharif* 2019	Parent	128.5	181.00	30.38	27.81	1.33	9	9	158	46	204	77.59	1.05	22.80
	Rabi 2019-20	Parent	131	139.00	29.15	28.80	1.38	10	11	182	44	226	80.54	1.06	22.08
	Kharif 2020	Parent	130	175.78	31.57	28.63	1.37	9	8	165	64	229	72.21	1.07	24.40
Vishnubhog Mutant V-17	*Kharif* 2019	M_4	109	120.25	22.13	30.45	0.75	37	26	77	49	126	61.10	1.34	11.11
	Rabi 2019-20	M_5	117.5	97.75	21.48	31.75	0.63	37	26	111	44	154	71.78	1.05	11.21
	Kharif 2020	M_6	112.5	111.85	23.08	33.30	0.62	28	20	70	47	116	60.00	1.52	12.71
Vishnubhog Mutant V-19-2	*Kharif* 2019	M_4	108	97.05	18.88	33.60	1.23	8	8	162	56	217	74.42	1.52	15.50
	Rabi 2019-20	M_5	112	82.00	17.60	28.95	1.38	14	13	132	41	173	76.20	1.35	19.54
	Kharif 2020	M_6	101	95.43	18.92	31.82	1.37	16	15	124	31	154	80.15	1.53	17.85
Vishnubhog Mutant V-74-6	*Kharif* 2019	M_4	107	111.33	25.45	33.63	1.56	8	7	197	44	241	81.91	1.44	25.55
	Rabi 2019-20	M_5	117	102.00	26.33	31.85	1.48	13	13	222	53	275	80.70	1.31	27.25
	Kharif 2020	M_6	101.5	109.58	24.35	33.33	1.52	12	9	170	42	212	80.14	1.31	27.36
Vishnubhog Mutant V-47	*Kharif* 2019	M_4	107.5	106.42	24.47	29.93	1.39	8	8	157	57	214	73.31	1.12	17.50
	Rabi 2019-20	M_5	116.5	96.50	23.81	29.15	1.36	15	15	141	42	183	77.06	1.06	19.00
	Kharif 2020	M_6	109	95.92	20.64	28.61	1.53	11	12	158	50	208	75.98	1.43	18.11
Vishnubhog Mutant V-45	*Kharif* 2019	M_4	96	103.50	26.40	34.13	2.08	9	9	176	47	223	78.91	1.38	21.50
	Rabi 2019-20	M_5	113	91.25	19.28	32.00	1.90	11	16	161	52	213	75.60	1.27	23.83
	Kharif 2020	M_6	102	107.75	23.63	33.40	2.00	9	8	171	58	229	74.85	1.33	22.87
Vishnubhog Mutant V-33	*Kharif* 2019	M_4	112	104.25	23.50	34.18	2.08	11	11	180	44	224	80.59	1.39	24.00
	Rabi 2019-20	M_5	118	104.50	24.25	33.03	1.35	14	13	166	48	214	77.75	1.41	23.77
	Kharif 2020	M_6	111	102.83	25.45	35.61	1.64	11	6	163	53	216	75.43	1.50	24.71
Vishnubhog Mutant V-45-2	*Kharif* 2019	M_4	106	129.00	24.20	41.25	1.49	10	10	161	61	222	72.50	1.44	18.25
	Rabi 2019-20	M_5	119	125.00	22.63	37.00	1.53	12	13	196	45	241	81.49	1.46	21.41
	Kharif 2020	M_6	112.5	128.45	24.93	36.48	1.53	9	7	212	53	265	80.08	1.48	20.62
Vishnubhog Mutant V-67	*Kharif* 2019	M_4	112.5	101.25	17.88	24.03	1.70	9	9	102	61	163	62.70	1.40	13.00
	Rabi 2019-20	M_5	119	77.00	18.15	18.05	1.28	11	11	88	53	141	62.42	1.47	11.07
	Kharif 2020	M_6	115	98.59	19.79	24.22	1.70	7	7	104	72	176	59.26	1.39	13.20
Vishnubhog	*Kharif* 2019	M_4	96	105.00	25.40	32.13	1.60	11	11	215	44	259	83.08	1.36	26.19

Mutant V-71-4	*Rabi* 2019-20	M_5	112	90.00	22.85	31.10	1.52	13	14	192	45	237	80.99	1.36	26.67
	Kharif 2020	M_6	97	105.25	22.38	31.59	1.50	11	10	192	33	224	85.36	1.81	26.17
Vishnubhog Mutant V-80	*Kharif* 2019	M_4	94	115.50	24.72	27.08	1.55	9	9	189	44	233	81.09	1.37	20.88
	Rabi 2019-20	M_5	113	108.45	23.80	28.05	1.40	12	10	187	47	234	79.88	1.18	21.68
	Kharif 2020	M_6	95.5	115.10	24.94	26.57	1.60	10	9	185	45	230	80.60	1.35	21.22
Vishnubhog Parent	*Kharif* 2019	Parent	121	147.25	25.10	36.83	1.57	9	9	165	49	214	77.31	1.23	18.50
	Rabi 2019-20	Parent	125	133.50	23.85	32.50	1.47	11	14	163	44	207	78.93	1.36	20.87
	Kharif 2020	Parent	118.5	150.33	23.58	42.08	1.56	8	7	163	63	226	72.03	1.44	19.20
Jhilli Mutant J-2-13	*Kharif* 2019	M_4	87	104.75	28.25	35.00	1.43	10	9	132	51	183	72.06	1.81	20.75
	Rabi 2019-20	M_5	107	101.58	25.50	36.13	1.51	10	15	120	49	169	71.72	1.86	18.47
	Kharif 2020	M_6	87	100.09	23.95	33.53	1.52	9	8	101	37	138	73.65	1.86	19.40
Jhilli Mutant J-12-1	*Kharif* 2019	M_4	86.5	107.00	29.00	28.50	1.63	10	9	176	45	221	79.58	2.20	23.95
	Rabi 2019-20	M_5	113	100.63	20.68	33.85	1.58	11	10	150	41	190	78.72	2.12	21.06
	Kharif 2020	M_6	88	97.49	26.16	28.28	1.49	10	9	159	30	189	83.95	2.31	24.30
Jhilli Mutant J-13-2	*Kharif* 2019	M_4	89	107.75	19.20	32.75	1.70	13	12	172	51	223	77.31	1.26	21.45
	Rabi 2019-20	M_5	113.5	101.45	22.83	31.78	1.59	11	9	147	49	195	75.14	1.21	18.58
	Kharif 2020	M_6	93.5	100.88	22.95	33.58	1.53	8	7	154	48	202	76.44	1.21	19.90
Jhili Dhan J-13-5	*Kharif* 2019	M_4	89.5	111.75	28.00	34.50	1.48	10	10	191	42	233	81.97	2.40	25.83
	Rabi 2019-20	M_5	108	99.50	24.08	31.03	1.46	13	14	151	34	185	81.48	1.65	27.08
	Kharif 2020	M_6	89	109.13	25.57	32.69	1.45	11	10	162	35	197	82.57	2.31	25.91
Jhilli Mutant J-15-1	*Kharif* 2019	M_4	86.5	105.75	25.25	33.00	1.39	12	12	182	48	230	79.32	2.34	23.70
	Rabi 2019-20	M_5	109.5	91.75	24.55	29.10	1.47	13	14	152	40	192	79.16	2.10	23.56
	Kharif 2020	M_6	90.5	102.80	23.78	29.55	1.55	9	8	169	40	209	80.68	2.48	25.01
Jhilli Dhan Parent	*Kharif* 2019	Parent	116	168.25	27.50	36.25	1.48	10	10	157	49	206	76.46	2.10	21.25
	Rabi 2019-20	Parent	124	142.75	24.05	32.95	1.47	11	9	150	49	198	75.54	1.67	20.52
	Kharif 2020	Parent	117.5	170.67	23.21	36.48	1.48	9	9	152	31	183	83.04	2.24	20.80
Dubraj selection -1	*Kharif* 2019	Check 1	115	150.50	22.48	37.40	1.25	8	7	126	37	163	77.34	1.73	19.01
	Rabi 2019-20	Check 1	129	133.50	22.48	33.31	1.33	10	9	152	44	196	77.77	1.63	18.75
	Kharif 2020	Check 1	110	152.93	22.65	36.65	1.28	9	8	152	60	212	71.75	1.94	18.25
Vishnubhog Selection-1	*Kharif* 2019	Check 2	114	148.25	26.35	29.15	1.30	11	10	150	49	199	75.34	1.34	20.87
	Rabi 2019-20	Check 2	130.5	136.00	24.00	32.95	1.37	11	11	156	45	201	77.55	1.35	21.04
	Kharif 2020	Check 2	109	149.75	23.98	32.60	1.33	11	10	154	55	209	73.87	1.50	20.25
Rajeshwari	*Kharif* 2019	Check 3	94.5	123.00	25.50	33.00	1.59	11	10	164	29	192	85.17	3.05	27.40
	Rabi 2019-20	Check 3	104	114.00	24.00	30.50	1.50	12	10	179	32	211	84.81	3.19	28.42
	Kharif 2020	Check 3	94.5	123.50	25.00	32.50	1.57	11	10	172	36	208	82.64	3.18	28.09

Supplementary Table S3: Mean performance of 24 rice genotypes for 18 grain quality traits taken during three consecutive seasons/ generations

Genotypes	Season/Year	Generation	Hul%	Mil%	HRR%	PadL	PadB	BRL	BRB	KL	KB	KLBR	CRL	CRW	ER	ASV	GC	AC%	Aroma
Samundchini Mutant S-49	*Kharif* 19	M_4	73.66	64.51	58.31	6.81	2.25	5.26	1.80	4.85	1.65	2.97	6.25	2.85	1.29	4	62.5	21.0	High
	*Rabi*19-20	M_5	72.36	64.01	57.81	6.80	2.25	5.25	1.80	4.90	1.65	2.97	6.20	2.80	1.27	4	62.5	20.3	High
	Kharif 20	M_6	74.44	67.06	54.95	6.80	2.20	5.20	1.80	4.60	1.60	2.89	5.40	2.80	1.17	4	60.5	23.8	High
Samundchini Mutant S-18-1	*Kharif* 19	M_4	78.84	69.98	61.30	6.29	2.45	4.20	1.79	3.85	1.70	2.26	7.81	2.63	2.03	3	52.5	23.5	Mild
	*Rabi*19-20	M_5	80.04	71.48	65.30	6.20	2.45	4.15	1.75	3.85	1.70	2.26	7.80	2.60	2.03	3	55.0	23.8	Mild
	Kharif 20	M_6	84.76	71.56	62.20	6.20	2.40	4.40	2.00	4.10	1.80	2.28	5.00	2.40	1.22	2	46.5	24.2	Mild
Samundchini Mutant S-50	*Kharif* 19	M_4	78.29	63.89	57.45	5.81	2.40	4.83	2.11	4.50	1.95	2.31	7.43	3.06	1.65	4	57.0	25.0	Mild
	*Rabi*19-20	M_5	78.51	63.89	59.95	5.80	2.40	4.85	2.05	4.50	1.95	2.31	7.40	3.00	1.64	4	57.0	24.5	Mild
	Kharif 20	M_6	73.78	63.16	55.16	5.80	2.40	4.80	1.80	3.80	1.75	2.17	6.00	2.60	1.58	2	45.5	23.7	Mild
Samundchini Parent	*Kharif* 19	P	77.97	67.05	57.20	6.36	2.41	5.81	2.05	4.88	2.01	2.43	8.22	2.83	1.69	4	59.0	24.5	Mild
	*Rabi*19-20	P	77.35	67.05	60.20	6.40	2.40	5.80	2.05	4.90	2.00	2.45	8.20	2.80	1.67	4	60.0	23.0	Mild
	Kharif 20	P	75.40	60.90	54.97	6.40	2.40	4.60	2.20	4.15	2.05	2.03	6.00	2.80	1.45	3	42.5	24.1	Mild
Vishnubhog Mutant V-17	*Kharif* 19	M_4	78.73	64.88	57.22	6.72	2.59	4.68	2.05	4.13	2.11	1.93	6.98	3.21	1.69	4	56.0	18.6	High
	*Rabi*19-20	M_5	78.07	67.38	59.22	6.80	2.55	4.65	2.05	4.05	2.10	1.93	7.00	3.20	1.73	4	57.5	19.1	High
	Kharif 20	M_6	76.30	71.21	58.08	6.80	2.60	5.00	2.20	4.20	1.95	2.16	6.40	2.60	1.52	4	47.5	23.5	High
Vishnubhog Mutant V-19-2	*Kharif* 19	M_4	79.38	68.69	60.99	6.46	2.75	4.41	2.41	2.62	2.28	1.15	6.43	3.03	3.43	3	75.5	24.3	Mild
	*Rabi*19-20	M_5	78.79	68.69	60.84	6.40	2.75	4.40	2.40	4.05	2.25	1.80	6.40	3.00	1.58	3	76.5	22.8	Mild
	Kharif 20	M_6	73.90	61.46	51.17	6.40	2.80	4.40	2.20	4.00	2.10	1.90	7.20	3.00	1.80	3	87.5	20.0	Mild
Vishnubhog Mutant V-74-6	*Kharif* 19	M_4	74.83	68.85	69.60	6.41	2.61	4.56	2.38	4.26	2.26	1.88	6.78	3.03	1.59	5	56.5	24.4	Mild
	*Rabi*19-20	M_5	77.80	68.85	65.10	6.40	2.60	4.55	2.35	4.45	2.25	1.98	6.80	3.00	1.53	5	59.0	23.9	Mild
	Kharif 20	M_6	81.56	65.97	60.11	6.40	2.60	4.20	2.00	4.20	1.90	2.21	6.00	3.20	1.43	5	57.5	26.2	Mild
Vishnubhog Mutant V-47	*Kharif* 19	M_4	72.17	66.55	61.29	6.53	2.60	4.56	2.08	4.38	1.98	2.23	6.98	3.06	1.59	2	64.0	24.4	Absent
	*Rabi*19-20	M_5	74.55	67.55	59.29	6.50	2.60	4.55	2.05	4.35	1.95	2.23	7.00	3.00	1.61	2	64.0	23.9	Absent
	Kharif 20	M_6	68.62	60.70	54.78	6.60	2.60	4.20	2.00	4.00	1.75	2.29	5.20	3.00	1.30	2	57.0	21.6	Absent
Vishnubhog Mutant V-45	*Kharif* 19	M_4	78.19	67.50	56.87	6.00	2.85	4.27	2.55	4.13	2.38	1.74	7.58	3.23	1.84	2	68.5	24.0	Mild
	*Rabi*19-20	M_5	73.82	66.00	56.87	6.00	2.85	4.20	2.50	4.00	2.40	1.67	7.60	3.20	1.90	2	67.5	23.5	Mild
	Kharif 20	M_6	76.21	65.65	58.25	6.00	2.80	4.00	2.00	3.75	1.80	2.09	5.40	3.20	1.44	2	42.5	23.6	Mild
Vishnubhog Mutant V-33	*Kharif* 19	M_4	73.39	68.88	61.34	6.06	3.15	4.16	2.38	4.21	2.23	1.87	6.63	3.23	1.58	5	59.0	28.9	Mild
	*Rabi*19-20	M_5	75.22	68.88	61.34	6.00	3.15	4.15	2.35	4.20	2.25	1.87	6.60	3.20	1.57	5	53.0	27.9	Mild
	Kharif 20	M_6	79.08	69.06	60.66	6.00	3.20	4.40	2.60	4.05	2.50	1.62	5.80	3.00	1.43	5	36.0	28.2	Mild
Vishnubhog Mutant V-45-2	*Kharif* 19	M_4	71.18	63.62	59.34	5.33	2.69	3.90	2.43	3.96	2.13	1.88	7.03	2.83	1.78	3	70.5	24.4	Absent
	*Rabi*19-20	M_5	71.77	63.62	57.84	5.20	2.65	3.90	2.35	3.95	2.10	1.88	7.00	2.80	1.77	3	69.0	23.9	Absent
	Kharif 20	M_6	74.12	64.47	58.82	5.20	2.60	4.00	2.20	3.60	2.00	1.80	6.60	3.00	1.84	3	55.0	23.4	Absent
Vishnubhog Mutant V-67	*Kharif* 19	M_4	68.76	61.73	53.65	5.51	3.25	4.01	2.06	3.84	1.95	1.97	6.66	3.05	1.73	2	96.5	18.4	Mild
	*Rabi*19-20	M_5	70.76	61.73	54.15	5.40	3.25	4.00	2.05	3.80	1.90	2.01	6.60	3.00	1.74	2	96.5	19.0	Mild
	Kharif 20	M_6	75.31	66.25	54.37	5.40	3.20	3.80	2.00	3.40	1.90	1.79	6.00	3.00	1.76	2	91.5	20.3	Mild
Vishnubhog Mutant V-71-4	*Kharif* 19	M_4	79.88	70.63	63.32	6.41	2.86	4.78	2.21	4.21	2.13	1.98	7.23	3.03	1.72	5	62.5	25.8	Mild

	*Rabi*19-20	M_5	77.51	67.13	59.82	6.40	2.85	4.75	2.20	4.20	2.10	2.00	7.20	3.00	1.71	5	57.5	25.0	Mild
	Kharif 20	M_6	72.23	65.32	55.91	6.40	2.80	4.60	2.00	4.20	1.90	2.21	7.20	3.00	1.71	5	52.0	23.5	Mild
Vishnubhog Mutant V-80	*Kharif* 19	M_4	79.25	70.08	66.64	6.16	2.00	4.43	2.00	4.08	1.83	2.23	5.18	2.48	1.27	4	36.0	27.2	Strong
	*Rabi*19-20	M_5	78.79	70.58	65.14	6.10	2.00	4.45	1.95	4.05	1.85	2.19	5.20	2.40	1.28	5	37.0	26.4	Strong
	Kharif 20	M_6	73.85	65.53	56.69	6.00	2.20	4.40	2.00	4.00	1.80	2.23	5.20	2.40	1.30	4	33.5	27.6	Strong
Vishnubhog Parent	*Kharif* 19	P	76.62	66.44	55.41	5.83	2.80	4.56	2.21	4.43	2.12	2.09	6.58	3.03	1.49	4	61.0	24.4	Low
	*Rabi*19-20	P	76.81	63.94	54.41	5.80	2.80	4.60	2.20	4.45	2.05	2.17	6.60	3.00	1.48	4	59.0	23.5	Low
	Kharif 20	P	74.31	62.32	54.72	5.80	2.80	4.40	2.40	4.20	2.20	1.91	5.40	3.00	1.29	4	53.5	22.2	Low
Jhilli Mutant J-2-13	*Kharif* 19	M_4	79.88	72.30	63.68	8.71	2.49	6.76	1.96	6.43	1.88	3.43	8.21	2.48	1.23	6	90.0	18.5	Absent
	*Rabi*19-20	M_5	79.90	71.80	62.68	8.80	2.45	6.80	1.95	6.45	1.85	3.49	8.20	2.40	1.23	6	92.5	17.9	Absent
	Kharif 20	M_6	70.69	63.33	54.45	8.80	2.40	6.20	1.95	6.41	1.75	3.66	8.00	3.00	1.60	6	75.0	18.6	Absent
Jhilli Mutant J-12-1	*Kharif* 19	M_4	77.97	68.90	58.03	7.61	2.25	6.46	2.01	6.28	1.88	3.38	7.26	2.83	1.16	4	66.5	27.1	Mild
	*Rabi*19-20	M_5	78.00	67.40	56.53	7.60	2.25	6.45	1.95	6.25	1.85	3.38	7.20	2.80	1.15	4	62.5	26.2	Mild
	Kharif 20	M_6	72.50	61.48	54.39	7.60	2.20	6.40	2.00	6.29	1.80	3.49	8.60	3.00	1.16	5	45.0	24.5	Mild
Jhilli Mutant J-13-2	*Kharif* 19	M_4	73.61	64.81	55.17	7.29	2.61	5.41	2.22	5.48	2.06	2.64	6.96	2.63	1.27	1	63.5	26.5	Absent
	*Rabi*19-20	M_5	73.70	63.31	56.67	7.20	2.60	5.40	2.15	5.40	2.05	2.64	7.00	2.60	1.30	1	65.0	26.3	Absent
	Kharif 20	M_6	76.31	68.95	57.76	7.20	2.60	5.00	2.00	5.42	2.02	2.68	6.80	3.00	1.62	1	45.5	24.3	Absent
Jhili Dhan J-13-5	*Kharif* 19	M_4	83.55	75.65	60.71	8.92	2.35	6.44	2.05	6.01	1.83	3.16	8.05	2.55	1.39	5	47.5	26.1	Absent
	*Rabi*19-20	M_5	81.81	72.65	58.71	9.00	2.35	6.40	2.05	6.02	1.80	3.22	8.00	2.60	1.38	5	47.5	24.4	Absent
	Kharif 20	M_6	77.87	63.70	56.53	9.00	2.40	6.60	2.00	6.00	1.80	2.90	7.60	3.20	1.46	5	50.0	24.4	Absent
Jhilli Mutant J-15-1	*Kharif* 19	M_4	75.65	67.64	58.23	9.40	2.45	6.91	1.80	6.28	1.68	3.79	9.03	2.35	1.44	5	42.5	26.5	Absent
	*Rabi*19-20	M_5	77.53	68.64	56.73	9.40	2.45	6.85	1.80	6.25	1.65	3.79	9.00	2.40	1.44	5	38.0	26.0	Absent
	Kharif 20	M_6	78.24	69.75	58.61	9.40	2.40	7.40	2.00	6.00	1.75	3.43	8.60	2.80	1.43	5	42.5	21.6	Absent
Jhilli Dhan Parent	*Kharif* 19	P	77.77	68.91	57.21	8.61	2.41	6.69	2.05	6.18	2.06	3.16	8.65	2.38	1.40	4	63.5	27.3	Absent
	*Rabi*19-20	P	78.22	67.91	58.71	8.60	2.40	6.20	2.00	6.15	1.95	3.16	8.60	2.40	1.40	4	61.0	26.8	Absent
	Kharif 20	P	75.43	65.27	56.28	8.60	2.40	6.00	2.00	5.40	1.85	2.94	7.00	2.40	1.30	4	42.5	27.4	Absent
Dubraj selection -1	*Kharif* 19	Ch1	76.28	66.60	53.18	8.05	2.41	6.22	2.15	5.96	2.11	2.88	9.48	3.03	1.59	4	60.0	26.9	High
	*Rabi*19-20	Ch1	76.28	67.60	54.68	8.00	2.40	6.15	2.15	5.90	2.05	2.88	9.40	3.00	1.59	4	57.5	26.7	High
	Kharif 20	Ch1	73.55	63.38	53.43	8.00	2.40	5.80	2.20	5.00	1.80	2.79	9.20	2.80	1.84	4	57.5	25.8	High
Vishnubhog Selection-1	*Kharif* 19	Ch2	74.47	67.14	52.40	6.40	2.30	4.96	2.18	4.75	2.11	2.27	7.43	3.23	1.56	5	63.5	25.5	Absent
	*Rabi*19-20	Ch2	74.47	65.14	53.90	6.40	2.30	4.90	2.15	4.65	2.05	2.27	7.40	3.20	1.59	5	65.0	25.3	Absent
	Kharif 20	Ch2	76.21	69.08	58.45	6.40	2.40	5.45	2.00	4.60	1.85	2.49	6.60	3.50	1.44	5	40.5	26.3	Absent
Rajeshwari	*Kharif* 19	Ch3	74.03	66.54	52.80	8.67	3.20	6.80	2.60	6.48	2.41	2.67	10.4	3.23	1.60	4	42.5	25.9	Absent
	*Rabi*19-20	Ch3	74.03	66.54	52.80	8.67	3.20	6.80	2.60	6.48	2.41	2.67	10.3	3.22	1.60	4	42.5	25.9	Absent
	Kharif 20	Ch3	74.02	66.53	52.8	8.66	3.2	6.8	2.6	6.47	2.40	2.66	10.4	3.24	1.62	4	42.5	25.9	Absent

Supplementary Table S4. Shapiro-Wilk W test of the agro-morphological and grain quality traits during three consecutive seasons.

Traits	N	M4 Kharif season 2019		M5 Rabi season 2019-20		M6 Kharif season 2020	
		Shapiro-Wilk W	p(normal)	Shapiro-Wilk W	p(normal)	Shapiro-Wilk W	p(normal)
DFF	24	0.925	**0.077**	0.956	**0.357**	0.951	**0.289**
PH	24	0.836	0.001	0.948	**0.243**	0.848	0.002
PL	24	0.947	**0.227**	0.944	**0.199**	0.960	**0.429**
FLL	24	0.963	**0.496**	0.906	**0.294**	0.958	**0.400**
FLW	24	0.896	0.018	0.764	0.000	0.787	0.000
TTP	24	0.462	0.000	0.482	0.000	0.560	0.000
ETP	24	0.551	0.000	0.807	0.000	0.667	0.000
FSP	24	0.932	**0.108**	0.977	**0.841**	0.943	**0.186**
SSP	24	0.955	**0.347**	0.950	**0.270**	0.958	**0.401**
TSP	24	0.912	**0.394**	0.980	**0.900**	0.962	**0.477**
SF%	24	0.888	0.012	0.914	0.042	0.878	0.008
HSW	24	0.870	0.005	0.815	0.001	0.910	0.035
GYP	24	0.947	**0.231**	0.925	**0.076**	0.972	**0.720**
Hul%	24	0.970	**0.669**	0.965	**0.538**	0.937	**0.139**
Mil%	24	0.969	**0.652**	0.969	**0.635**	0.957	**0.374**
PadL	24	0.888	0.012	0.895	0.017	0.897	0.019
PadB	24	0.944	**0.202**	0.936	**0.134**	0.864	0.004
BRL	24	0.878	0.008	0.888	0.012	0.904	0.026
BRB	24	0.950	**0.273**	0.959	**0.424**	0.793	0.000
KL	24	0.921	**0.063**	0.865	0.004	0.836	0.001
KB	24	0.971	**0.692**	0.963	**0.493**	0.841	0.002
KLBR	24	0.920	**0.057**	0.919	**0.056**	0.897	0.018
CRL	24	0.953	**0.312**	0.954	**0.322**	0.914	0.043
CRW	24	0.896	0.018	0.896	0.018	0.919	0.055
ER	24	0.873	0.006	0.874	0.006	0.930	**0.096**
ASV	24	0.941	**0.171**	0.941	**0.171**	0.942	**0.177**
GC	24	0.928	**0.086**	0.908	0.032	0.836	0.001
AC%	24	0.867	0.005	0.905	0.028	0.969	**0.653**
HRR%	24	0.955	**0.346**	0.956	**0.359**	0.976	**0.819**

Supplementary Table S5. Correlation coefficients for agro-morphological traits during all three seasons

Characters	Seasons	DFF	PH	PL	FLL	FLW	TTP	ETP	FSP	SSP	TSP	SF%	HSW	GYP
Days to 50% Flowering (DFF)	Kharif 2019	-	0.633**	0.047NS	-0.180NS	-0.300*	0.123NS	0.077NS	-0.318*	0.103NS	-0.305*	-0.243NS	-0.561**	-0.220NS
	Rabi 2019-20	-	0.678**	0.224NS	-0.016NS	-0.200NS	-0.035NS	-0.165NS	0.074NS	0.187NS	-0.256NS	-0.204NS	-0.463**	-0.266NS
	Kharif 2020	-	0.605**	0.316*	-0.051NS	-0.364*	0.216NS	0.157NS	0.040NS	0.606**	0.218NS	-0.428**	-0.556**	-0.138NS
Plant height (cm) (PH)	Kharif 2019	0.645**	-	0.364*	0.111NS	-0.231NS	-0.068NS	-0.085NS	-0.164NS	-0.168NS	-0.215NS	0.033NS	-0.068NS	-0.011*
	Rabi 2019-20	0.714**	-	0.477**	0.316*	-0.010NS	-0.192NS	-0.284NS	0.342*	-0.080NS	-0.096NS	0.188NS	-0.017NS	-0.136*
	Kharif 2020	0.624**	-	0.380**	0.314*	-0.123NS	-0.124NS	-0.143NS	0.175NS	0.303*	0.251NS	-0.047NS	-0.029NS	-0.036*
Panicle length (cm) (PL)	Kharif 2019	0.041NS	0.380**	-	-0.163NS	-0.009NS	-0.128NS	-0.121NS	0.357*	-0.376**	0.273NS	0.465**	0.072NS	0.630*
	Rabi 2019-20	0.227NS	0.496**	-	-0.145NS	-0.224NS	-0.023NS	-0.011NS	0.535**	-0.144NS	0.159NS	0.372**	-0.210NS	0.418*
	Kharif 2020	0.347*	0.419**	-	-0.272NS	-0.264NS	0.068NS	-0.001NS	0.393**	0.143NS	0.404**	0.166NS	-0.233NS	0.625**
Flag leaf length(FLL) (cm)	Kharif 2019	-0.204NS	0.120NS	-0.160NS	-	0.218NS	-0.228NS	-0.210NS	0.114NS	0.011NS	0.122NS	0.125NS	0.360*	0.085NS
	Rabi 2019-20	-0.052NS	0.327*	-0.160NS	-	0.394**	-0.183NS	-0.028NS	0.140NS	-0.025NS	0.138NS	0.154NS	0.283NS	0.164NS
	Kharif 2020	-0.062NS	0.333*	-0.280NS	-	0.195NS	-0.184NS	-0.245NS	-0.070NS	-0.065NS	-0.084NS	0.043NS	0.274NS	0.172NS
Flag leaf width(FLW) (cm)	Kharif 2019	-0.301*	-0.241NS	-0.003NS	0.237NS	-	-0.698**	-0.670**	0.516**	-0.121NS	0.506**	0.441**	0.177NS	0.299*
	Rabi 2019-20	-0.216NS	-0.011NS	-0.229NS	0.405**	-	-0.852**	-0.486**	0.370**	0.077NS	0.583**	0.494**	0.383**	0.455**
	Kharif 2020	-0.388**	-0.139NS	-0.280NS	0.241NS	-	-0.821**	-0.790**	0.366*	0.110NS	0.369**	0.282NS	0.260NS	0.140NS
Total tillers/ plant (TTP)	Kharif 2019	0.116NS	-0.070NS	-0.134NS	-0.254NS	-0.714**	-	0.986**	-0.502**	0.070NS	-0.505**	-0.463**	-0.221NS	0.278NS
	Rabi 2019-20	-0.044NS	-0.199NS	-0.014NS	-0.193NS	-0.878**	-	0.640**	-0.429**	-0.027NS	-0.463**	-0.473**	-0.314*	0.414**
	Kharif 2020	0.232NS	-0.131NS	0.065NS	-0.186NS	-0.922**	-	0.955**	-0.516**	-0.229NS	-0.542**	-0.298*	-0.223NS	0.221NS
Effective tillers/plant (ETP)	Kharif 2019	0.073NS	-0.087NS	-0.131NS	-0.234NS	-0.691**	0.999**	-	-0.448**	0.065NS	-0.449**	-0.421**	-0.194NS	0.243NS
	Rabi 2019-20	-0.149NS	-0.398**	-0.052NS	0.000NS	-0.677**	0.918**	-	-0.235NS	0.148NS	-0.323*	-0.442**	-0.294*	0.287*
	Kharif 2020	0.175NS	-0.156NS	-0.018NS	-0.268NS	-0.900**	0.990**	-	-0.498**	-0.285*	-0.542**	-0.228NS	-0.184NS	0.227NS
Fertile spikelets/ panicle(FSP)	Kharif 2019	-0.335*	-0.166NS	0.375**	0.133NS	0.549**	-0.533**	-0.474**	-	-0.283NS	0.968**	0.858**	0.117NS	0.730**
	Rabi 2019-20	-0.269NS	0.156NS	0.444**	0.187NS	0.497**	-0.421**	-0.339*	-	0.817**	-0.473**	0.791**	0.971**	0.308*
	Kharif 2020	-0.151NS	0.046NS	0.631**	-0.193NS	0.198NS	-0.253NS	-0.272NS	-	0.767**	-0.235NS	0.644**	0.761**	0.166**
Sterile spikelets/ panicle(SSP)	Kharif 2019	0.132NS	-0.191NS	-0.472**	0.056NS	-0.083NS	0.068NS	0.105NS	-0.397**	-	-0.034NS	-0.696**	-0.400**	-0.598**
	Rabi 2019-20	0.483**	-0.181NS	-0.294*	-0.076NS	0.153NS	-0.017NS	-0.390**	-0.179NS	-	-0.053NS	-0.265NS	-0.374**	-0.309**
	Kharif 2020	0.665**	0.326*	0.163NS	-0.045NS	0.098NS	-0.241NS	-0.317*	0.114NS	-	0.413**	-0.644**	-0.507**	-0.211**
Total spikelets/ panicle(TSP)	Kharif 2019	-0.328*	-0.222NS	0.292*	0.155NS	0.567**	-0.554**	-0.483**	0.977**	-0.192NS	-	0.712**	0.017NS	0.605**
	Rabi 2019-20	-0.271NS	-0.100NS	0.193NS	0.139NS	0.611**	-0.520**	-0.448**	0.660**	-0.014NS	-	0.712**	-0.015NS	0.718**
	Kharif 2020	0.227NS	0.259NS	0.454**	-0.065NS	0.394**	-0.562**	-0.580**	0.959**	0.390**	-	0.403**	-0.201NS	0.598**
Spikelet fertility (SF%)	Kharif 2019	-0.263NS	0.032NS	0.504**	0.113NS	0.445**	-0.489**	-0.461**	0.903**	-0.710**	0.801**	-	0.300*	0.837**
	Rabi 2019-20	-0.241NS	0.195NS	0.408**	0.183NS	0.519**	-0.503**	-0.584**	0.753**	-0.488**	0.801**	-	0.290*	0.897**
	Kharif 2020	-0.457**	-0.047NS	0.202NS	0.039NS	0.334*	-0.320*	-0.251NS	0.680**	-0.619**	0.454**	-	0.356*	0.693**
Hundred Seed weight (g) (HSW)	Kharif 2019	-0.575**	-0.072NS	0.071NS	0.415**	0.183NS	-0.222NS	-0.199NS	0.120NS	-0.449**	0.024NS	0.302*	-	0.232*
	Rabi 2019-20	-0.496**	-0.015NS	-0.221NS	0.291*	0.384**	-0.320*	-0.392**	-0.028NS	-0.647**	-0.019NS	0.319*	-	0.288*
	Kharif 2020	-0.617**	-0.038NS	-0.232NS	0.290*	0.296*	-0.236NS	-0.198NS	-0.050NS	-0.593**	-0.215NS	0.406**	-	0.124*
Grain yield/plant (GYP) (g)	Kharif 2019	-0.228NS	-0.019*	0.696**	0.118NS	0.326*	0.297*	0.257NS	0.788**	-0.770**	0.663**	0.934**	0.268*	-
	Rabi 2019-20	-0.269NS	-0.156*	0.444**	0.187NS	0.497**	0.421**	0.339*	0.817**	-0.473**	0.791**	0.971**	0.308*	-
	Kharif 2020	-0.151NS	-0.046*	0.631**	0.193NS	0.198NS	0.253*	0.272NS	0.767**	-0.235*	0.644**	0.761**	0.166*	-

NB: Values below diagonal represents the genotypic correlation coefficients whereas above diagonal represents phenotypic correlation coefficients

Supplementary Table S6. Correlation coefficients for grain quality traits during all three seasons

Traits	Seasons	Hul (%)	Mil (%)	PadL	PadB	BRL	BRB	KL	KB	KLBR	CRL	CRW	ER	GC	AC (%)	HRR (%)
**Hul (%)**	Kharif 2019	-	0.696**	0.315*	-0.419**	0.291*	-0.182NS	0.137NS	-0.138NS	0.206NS	0.098NS	-0.292*	0.296*	-0.273NS	0.088NS	0.291*
	Rabi 2019-20	-	0.771**	0.339*	-0.421**	0.300*	-0.249NS	0.230NS	-0.191NS	0.276NS	0.097NS	-0.391**	0.312*	-0.279NS	0.070NS	0.470**
	Kharif 2020	-	0.657**	-0.041NS	0.061NS	-0.050NS	0.069NS	-0.098NS	0.052NS	-0.099NS	-0.198NS	-0.197NS	-0.104NS	-0.209NS	0.283NS	0.705**
**Mil (%)**	Kharif 2019	0.822**	-	0.420**	-0.256NS	0.325*	-0.120NS	0.254NS	-0.142NS	0.286*	0.111NS	-0.388**	0.349*	-0.199NS	0.227NS	0.497**
	Rabi 2019-20	0.833**	-	0.433**	-0.307*	0.290*	-0.194NS	0.238NS	-0.169NS	0.283NS	0.154NS	-0.390**	0.348*	-0.306*	0.067NS	0.507**
	Kharif 2020	0.664**	-	0.030NS	0.119NS	0.066NS	0.042NS	-0.054NS	0.007NS	-0.047NS	-0.087NS	-0.164NS	0.007NS	-0.258NS	0.273NS	0.722**
**PadL**	Kharif 2019	0.346*	0.536**	-	-0.226NS	0.922**	-0.247NS	0.823**	-0.205NS	0.855**	0.686**	-0.497**	0.810**	-0.241NS	0.177NS	-0.097NS
	Rabi 2019-20	0.480**	0.547**	-	-0.232NS	0.912**	-0.219NS	0.895**	-0.249NS	0.838**	0.681**	-0.491**	0.813**	-0.252NS	0.137NS	-0.175NS
	Kharif 2020	-0.106NS	0.019NS	-	-0.253NS	0.924**	-0.020NS	0.766**	-0.146NS	0.726**	0.733**	0.096NS	0.728**	-0.108NS	-0.013NS	-0.172NS
**PadB**	Kharif 2019	-0.482**	-0.329*	-0.223NS	-	-0.289*	0.593**	-0.215NS	0.586**	-0.410**	0.116NS	0.529**	-0.190NS	0.333*	-0.141NS	-0.219NS
	Rabi 2019-20	-0.530**	-0.361*	-0.234NS	-	-0.282NS	0.613**	-0.225NS	0.589**	-0.445**	0.118NS	0.528**	-0.190NS	0.261NS	-0.078NS	-0.271NS
	Kharif 2020	0.016NS	0.120NS	-0.262NS	-	-0.295*	0.600**	-0.235NS	0.651**	-0.512**	0.053NS	0.410**	-0.142NS	0.253NS	-0.093NS	-0.028NS
**BRL**	Kharif 2019	0.332*	0.401**	0.940**	-0.294*	-	-0.256NS	0.895**	-0.235NS	0.913**	0.722**	-0.463**	0.808**	-0.192NS	0.190NS	-0.212NS
	Rabi 2019-20	0.375**	0.342*	0.937**	-0.288*	-	-0.222NS	0.968**	-0.272NS	0.896**	0.707**	-0.445**	0.794**	-0.211NS	0.129NS	-0.225NS
	Kharif 2020	-0.106NS	0.082NS	0.932**	-0.317*	-	0.007NS	0.872**	-0.114NS	0.809**	0.786**	0.142NS	0.756**	-0.219NS	0.049NS	-0.177NS
**BRB**	Kharif 2019	-0.212NS	-0.131NS	-0.262NS	0.640**	-0.271NS	-	-0.223NS	0.908**	-0.512**	0.074NS	0.584**	-0.251NS	0.067NS	0.241NS	-0.086NS
	Rabi 2019-20	-0.424**	-0.251NS	-0.229NS	0.679**	-0.240NS	-	-0.158NS	0.946**	-0.549**	0.113NS	0.596**	-0.269NS	0.023NS	0.203NS	-0.206NS
	Kharif 2020	0.064NS	0.024NS	-0.029NS	0.714**	-0.009NS	-	0.099NS	0.905**	-0.327*	0.261NS	0.308*	0.124NS	-0.138NS	0.193NS	-0.048NS
**KL**	Kharif 2019	0.120NS	0.261NS	0.885**	-0.262NS	0.968**	-0.248NS	-	-0.216NS	0.861**	0.664**	-0.411**	0.742**	-0.105NS	0.196NS	-0.245NS
	Rabi 2019-20	0.272NS	0.294*	0.924**	-0.233NS	0.976**	-0.176NS	-	-0.206NS	0.890**	0.691**	-0.435**	0.778**	-0.138NS	0.187NS	-0.242NS
	Kharif 2020	-0.143NS	-0.087NS	0.777**	-0.262NS	0.878**	0.097NS	-	-0.013NS	0.878**	0.756**	0.204NS	0.690**	-0.244NS	0.136NS	-0.219NS
**KB**	Kharif 2019	-0.173NS	-0.136NS	-0.239NS	0.662**	-0.247NS	0.987**	-0.242NS	-	-0.544**	0.111NS	0.616**	-0.238NS	0.104NS	0.176NS	-0.143NS
	Rabi 2019-20	-0.373**	-0.220NS	-0.259NS	0.653**	-0.300*	0.974**	-0.233NS	-	-0.616**	0.085NS	0.643**	-0.310*	0.024NS	0.189NS	-0.129NS
	Kharif 2020	0.266NS	0.106NS	-0.180NS	0.866**	-0.141NS	0.977**	-0.024NS	-	-0.473**	0.151NS	0.346*	-0.003NS	-0.093NS	0.204NS	-0.046NS
**KLBR**	Kharif 2019	0.240NS	0.372**	0.893**	-0.428**	0.935**	-0.537**	0.941**	-0.541**	-	0.542**	-0.616**	0.754**	-0.140NS	0.090NS	-0.135NS
	Rabi 2019-20	0.382**	0.347*	0.871**	-0.466**	0.917**	-0.554**	0.906**	-0.612**	-	0.501**	-0.646**	0.764**	-0.125NS	0.057NS	-0.122NS
	Kharif 2020	-0.225NS	-0.122NS	0.770**	-0.620**	0.848**	-0.318*	0.909**	-0.429**	-	0.564**	-0.010NS	0.584**	-0.171NS	0.028NS	-0.146NS
**CRL**	Kharif 2019	0.111NS	0.151NS	0.704**	0.120NS	0.735**	0.077NS	0.730**	0.109NS	0.575**	-	-0.080NS	0.820**	-0.175NS	0.166NS	-0.408**
	Rabi 2019-20	0.139NS	0.196NS	0.702**	0.122NS	0.719**	0.113NS	0.701**	0.076NS	0.522**	-	-0.083NS	0.819**	-0.194NS	0.204NS	-0.311*

	Kharif 2020	-0.293*	-0.105^{NS}	0.743**	0.073^{NS}	0.798**	0.290*	0.774**	0.166^{NS}	0.603**	-	0.361*	0.867**	0.032^{NS}	-0.001^{NS}	-0.368*
**CRW**	Kharif 2019	-0.355*	-0.455**	-0.551**	0.573**	-0.494**	0.639**	-0.459**	0.743**	-0.686**	-0.108^{NS}	-	-0.598**	0.118^{NS}	-0.106^{NS}	-0.270^{NS}
	Rabi 2019-20	-0.497**	-0.488**	-0.545**	0.576**	-0.477**	0.674**	-0.476**	0.745**	-0.713**	-0.110^{NS}	-	-0.627**	0.087^{NS}	-0.037^{NS}	-0.299*
	Kharif 2020	0.010^{NS}	0.003^{NS}	0.120^{NS}	0.571**	0.177^{NS}	0.398**	0.228^{NS}	0.411**	0.005^{NS}	0.434**	-	-0.131^{NS}	0.089^{NS}	-0.041^{NS}	-0.189^{NS}
**ER**	Kharif 2019	0.307*	0.426**	0.858**	-0.201^{NS}	0.853**	-0.267^{NS}	0.821**	-0.275^{NS}	0.802**	0.847**	-0.621**	-	-0.165^{NS}	0.166^{NS}	-0.159^{NS}
	Rabi 2019-20	0.368*	0.405**	0.851**	-0.194^{NS}	0.810**	-0.289*	0.795**	-0.350*	0.795**	0.837**	-0.626**	-	-0.180^{NS}	0.171^{NS}	-0.074^{NS}
	Kharif 2020	-0.314*	-0.082^{NS}	0.768**	-0.156^{NS}	0.801**	0.161^{NS}	0.753**	0.006^{NS}	0.659**	0.901**	-0.008^{NS}	-	-0.039^{NS}	0.060^{NS}	-0.277^{NS}
**GC**	Kharif 2019	-0.342*	-0.352*	-0.267^{NS}	0.355*	-0.211^{NS}	0.072^{NS}	-0.223^{NS}	0.137^{NS}	-0.150^{NS}	-0.175^{NS}	0.211^{NS}	-0.186^{NS}	-	-0.513**	-0.129^{NS}
	Rabi 2019-20	-0.362*	-0.337*	-0.285*	0.296*	-0.230^{NS}	0.086^{NS}	-0.152^{NS}	0.103^{NS}	-0.160^{NS}	-0.206^{NS}	0.227^{NS}	-0.251^{NS}	-	-0.609**	-0.087^{NS}
	Kharif 2020	-0.359*	-0.397**	-0.114^{NS}	0.261^{NS}	-0.232^{NS}	-0.166^{NS}	-0.261^{NS}	-0.132^{NS}	-0.181^{NS}	0.028^{NS}	0.148^{NS}	-0.062^{NS}	-	-0.637**	-0.426**
**AC (%)**	Kharif 2019	0.157^{NS}	0.380**	0.193^{NS}	-0.159^{NS}	0.212^{NS}	0.246^{NS}	0.200^{NS}	0.144^{NS}	0.130^{NS}	0.171^{NS}	-0.133^{NS}	0.192^{NS}	-0.642**	-	0.014^{NS}
	Rabi 2019-20	0.089^{NS}	0.161^{NS}	0.170^{NS}	-0.090^{NS}	0.164^{NS}	0.208^{NS}	0.207^{NS}	0.181^{NS}	0.081^{NS}	0.214^{NS}	-0.134^{NS}	0.220^{NS}	-0.710**	-	0.014^{NS}
	Kharif 2020	0.528**	0.319*	-0.010^{NS}	-0.080^{NS}	0.066^{NS}	0.317*	0.178^{NS}	0.297*	0.041^{NS}	-0.027^{NS}	-0.081^{NS}	0.039^{NS}	-0.860**	-	0.357*
**HRR (%)**	Kharif 2019	0.353*	0.593**	-0.105^{NS}	-0.220^{NS}	-0.236^{NS}	-0.098^{NS}	-0.285*	-0.139^{NS}	-0.140^{NS}	-0.471**	-0.321*	-0.200^{NS}	-0.163^{NS}	0.038^{NS}	-
	Rabi 2019-20	0.778**	0.908**	-0.168^{NS}	-0.440**	-0.378**	-0.357*	-0.428**	-0.273^{NS}	-0.224^{NS}	-0.549**	-0.404**	-0.227^{NS}	-0.136^{NS}	-0.273^{NS}	-
	Kharif 2020	0.871**	0.644**	-0.261^{NS}	-0.119^{NS}	-0.246^{NS}	-0.078^{NS}	-0.292*	0.016^{NS}	-0.246^{NS}	-0.503**	-0.148^{NS}	-0.493**	-0.599**	0.453**	-

NB: Values below diagonal represents the genotypic correlation coefficients whereas above diagonal represents phenotypic correlation coefficients

Supplementary Table S7. Details of the three rice landraces used in the current study

S. No.	Accession No.	Name of rice landraces	Special feature(s)	Undesirable trait(s)	Origin
1	IC-125004	Vishnubhog	Aromatic short bold grains	Tall and late maturity, poor yield potential	Sarguja
2	IC-386390	Samundchini	Aromatic short slender grains	Tall and late maturity, poor yield potential	Bilaspur
3	IC-377273	Jhilli	Super fine grains/ long slender	Tall and late maturity, poor yield potential	Mahasamund
